# Supertwisted Chiral Gyroid Mesophase in Chiral Rod‐Like Compounds

**DOI:** 10.1002/anie.202403156

**Published:** 2024-05-02

**Authors:** Yan Wang, Shu‐Gui Yang, Ya‐Xin Li, Yu Cao, Feng Liu, Xiang‐Bing Zeng, Liliana Cseh, Goran Ungar

**Affiliations:** ^1^ State Key Laboratory for Mechanical Behaviour of Materials Shaanxi International Research Center for Soft Matter Xi'an Jiaotong University 710049 Xi'an China; ^2^ Department of Materials Science and Engineering University of Sheffield S1 3JD Sheffield UK; ^3^ School of Chemistry and Chemical Engineering Henan University of Technology 450001 Zhengzhou China; ^4^ Romanian Academy Coriolan Dragulescu Institute of Chemistry 300223 Timisoara Romania

**Keywords:** Liquid crystals, polycatenar, circular dichroism, depolarized fluorescence, thienofluorenone

## Abstract

Among the intriguing bicontinuous self‐assembled structures, the gyroid cubic is the most ubiquitous. It is found in block and star polymers, surfactants with or without solvent, in thermotropic liquid crystals with end‐ or side‐chains, and in biosystems providing structural color and modelling cell mitosis. It contains two interpenetrating networks of opposite chirality and is thus achiral if, as usual, the content of the two nets is the same. However, we now find that this is not the case for strongly chiral compounds. While achiral molecules follow the opposite twists of nets 1 and 2, molecules with a chiral center in their rod‐like core fail to follow the 70° twist between junctions in net 2 and instead wind against it by −110° to still match the junction orientation. The metastable chiral gyroid is a high‐entropy high‐heat‐capacity mesophase. The homochirality of its nets makes the CD signal of the thienofluorenone compounds close to that in the stable *I*23 phase with 3 isochiral nets.

## Graphical abstract

Molecules that cannot self‐assemble in either layers or cylinders often form complex in‐between “bicontinuous” structures made up of interpenetrating networks. The most widespread is the achiral “gyroid” phase, containing two antichiral networks. Here it is shown that the introduction of an asymmetric unit into the rod‐like molecular core can rewind the segments of one of the nets, “supertwisting” them in reverse and making the famous gyroid chiral.

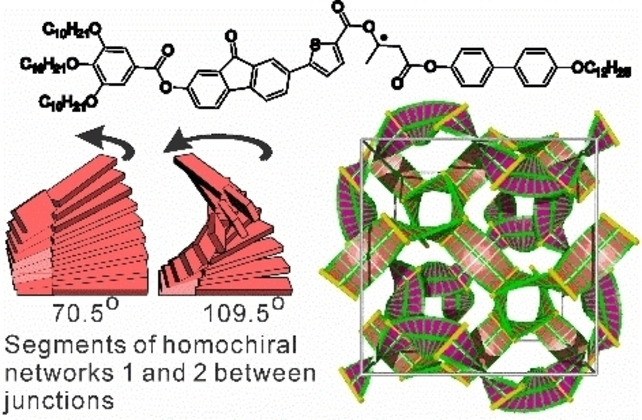

Bicontinuous phases are some of the most important and intriguing nanoscale structures produced by self‐assembly, found in a wide range of systems including both lyotropic^[1]^ and thermotropic^[2]^ liquid crystals (LC), amphiphiles, block and star polymers[^3^, ^4^] and dendrons.[^5^, ^6^] They can be used in photonics,[^7^, ^8^] as 3D electronic or ionic conductors,[^6^, ^9^] in membranes^[10]^ and as templates for well‐defined porous ceramics for use in separation or catalysis.^[11]^ The most well know and prominent among these phases is the gyroid cubic, consisting of two infinite interpenetrating networks.[^2^, ^12^] When it is formed by rod‐like mesogens, the orientation of the molecules follows the helical trajectories of the two networks. But since the nets are of opposite helical sense, there is no net chirality. However, one may ask what would happen if the molecular rods themselves are chiral? Here we show that in that case the molecular train in one of the two networks inverts its twist sense and follows a tighter “supertwisted” helix, making both networks homochiral and thus the gyroid optically active.

Molecules with a rigid or semirigid rod‐like core bearing pendant end‐chains are typical mesogens. With one flexible chain at one or both ends they usually form nematic or smectic LCs. With two or more end‐chains these so‐called polycatenars may form either a network‐based LC phase with 3D long‐range order[^13^, ^14^, ^15^, ^16^, ^17^, ^18^, ^19^] or a columnar phase with 2D[^20^, ^21^, ^22^, ^23^] and sometimes 3D order.[^24^, ^25^, ^26^] The 3D network phases are formed of 2[^2^, ^12^] or 3[^27^, ^28^] infinite interpenetrating nets—see Figure 1. The network segments are columns made up of stacked “rafts”, each containing 2–4 molecules lying side‐by‐side perpendicular to the column axis (Figure 1c). Three such phases are known in polycatenars: (i) the “double gyroid” cubic, spacegroup $Ia\bar{3}d$ , with two networks separated by the G‐type surface of minimum curvature;[^2^, ^12^, ^29^] (ii) the triple‐network cubic, spacegroup *I*23,[^28^, ^30^] and (iii) the tetragonal 2‐network “Smectic‐Q” phase (*I*4_1_22).^[31]^ The network segments are joined by 3‐way ($Ia\bar{3}d$ and *I*23) or 4‐way junctions (*I*4_1_22). A fourth phase, closely related to the gyroid, has also been reported recently, described alternatively as tetragonal^[32]^ and orthorhombic,^[33]^ possibly requiring further elucidation.

**Figure 1:**
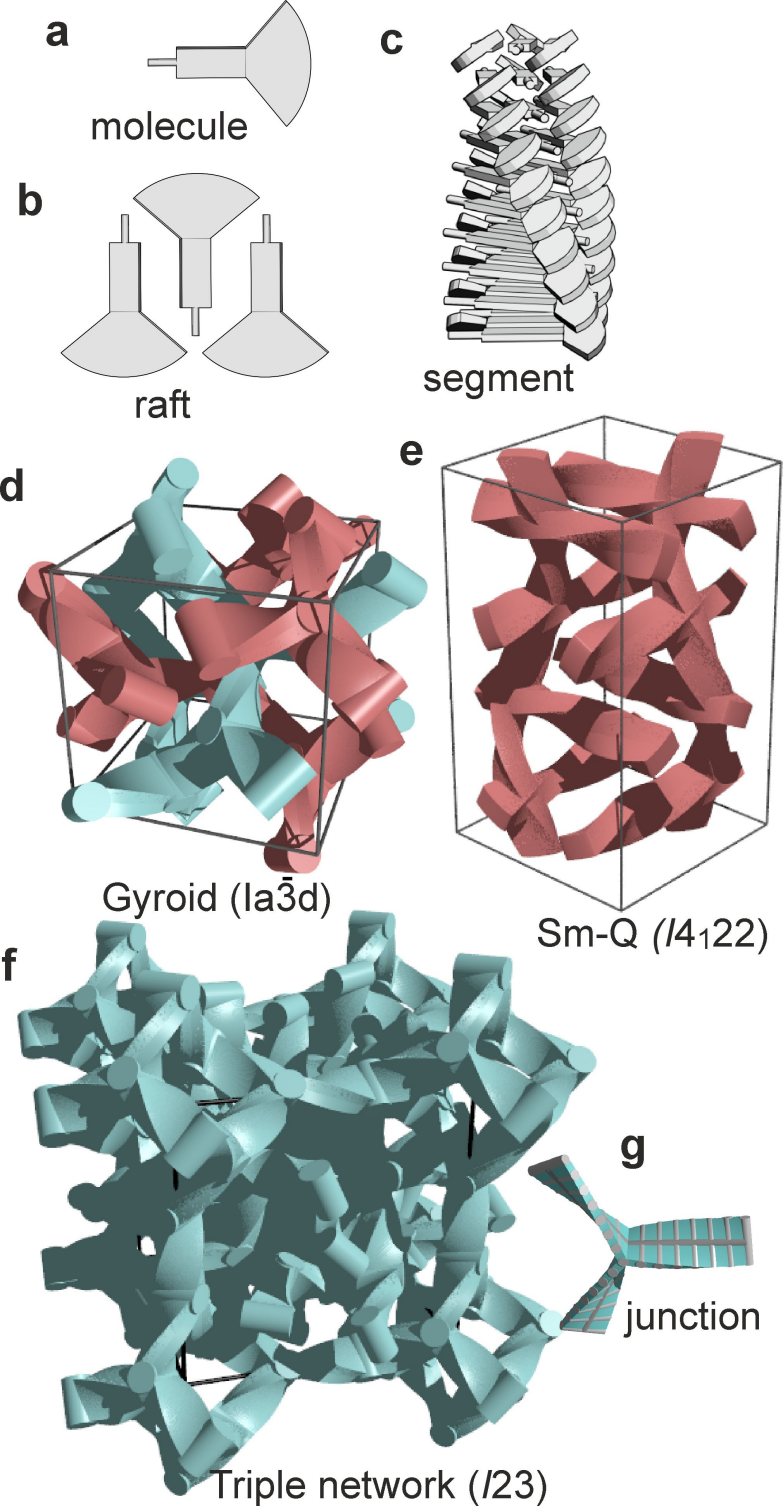
(a) A schematic polycatenar molecule with the fan symbolizing multiple end‐chains. (b) A “raft” of three molecules. (c) A helical columnar segment between two junctions of a network LC phase consisting of stacked rafts. (d–f) Packing of helical segments in the three bicontinuous network mesophases in polycatenars: gyroid cubic, Smectic‐Q and Triple net cubic. Red and blue colours indicate left and right twist. (g) A 3‐way junction; ribbons represent segments with ribs symbolizing orientation of molecular rods. Each rib represents a raft.

It was not until 2014 that it emerged that the triple‐network phase is always optically active, hence chiral, even when containing no chiral molecules.^[34]^ The same was later found for the Smectic‐Q.^[30]^ This led to the concept of twisted network segments in which the stacked rafts are rotated consistently in the same sense by a small angle, 7–10°, relative to neighbouring rafts. The twist alleviates clash between bulky end‐chains. It was argued that what maintains the long‐range helical sense throughout the infinite net is the smooth confluence of the 3 or 4 branches at a junction if all branches are homochiral.

Here we examine what might happen if the compounds are intrinsically chiral and, moreover, strongly chiral, with the asymmetric center in the rod‐like mesogen and not just in the pendant chain, as in the majority of chiral LCs. In the latter case “helix may disregard chirality”.^[35]^ Thus tetracatenar thienofluorenone‐containing compounds **1*R*** and **1*S*** were prepared, with either an *R* or *S* hydroxybutyrate linkage within the rod‐like core (Scheme 1). Recently we found that similar chiral compounds bearing six chains form a LC phase of coexisting straight right‐ and left‐handed helical columns, spacegroup *Fddd*.^[26]^ However, the *Fddd* formed only if both *R* and *S* enantiomers were present and not in pure enantiomers. Thus a similar situation may be expected in the gyroid cubic, with its coexisting right and left‐handed networks, since in a pure enantiomer 50 % of molecules would have to enter the network of “wrong” chirality.

**Scheme 1:**
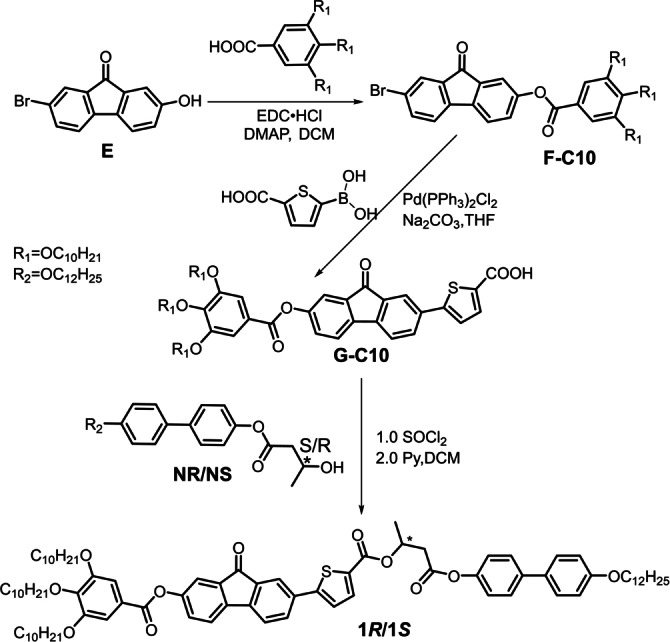
Synthesis of compounds **1*S*** and **1*R***.

The synthesis of **1*R*** and **1*S*** is shown in Scheme 1. The detailed procedures and analytical data, as well as description of all methods, are given in Supporting Information.

The crystalline compounds **1** melt at 89 °C into a LC phase with no birefringence, which turns to isotropic (Iso) liquid at 127 °C. X‐ray diffraction (XRD) shows the phase to be the triple network cubic *I*23, shown in Figure 1f—see also Figures S2, S4 and Table S2 in Supporting Information. When cooled from Iso relatively fast (>10 K/min) the same mesophase appears at a significantly lower temperature. The tendency for these pure enantiomers to form the *I*23 phase is not surprising since all three networks in it are isochiral.^[28]^ However, on slower cooling another non‐birefringent phase forms first. Synchrotron XRD traces recorded during 2 K/min cooling, displayed in Figure 2a, show this to have the main features of the $Ia\bar{3}d$ gyroid, dominated as usual by the strongest two Bragg reflections (211) and (220). However on further cooling the phase transforms to the *I*23. Isothermal XRD experiments were also conducted, with the time‐resolved traces at 121 °C shown in Figure 2b. Here the gyroid is seen to form first, but is replaced by the *I*23 after 3 minutes.

**Figure 2:**
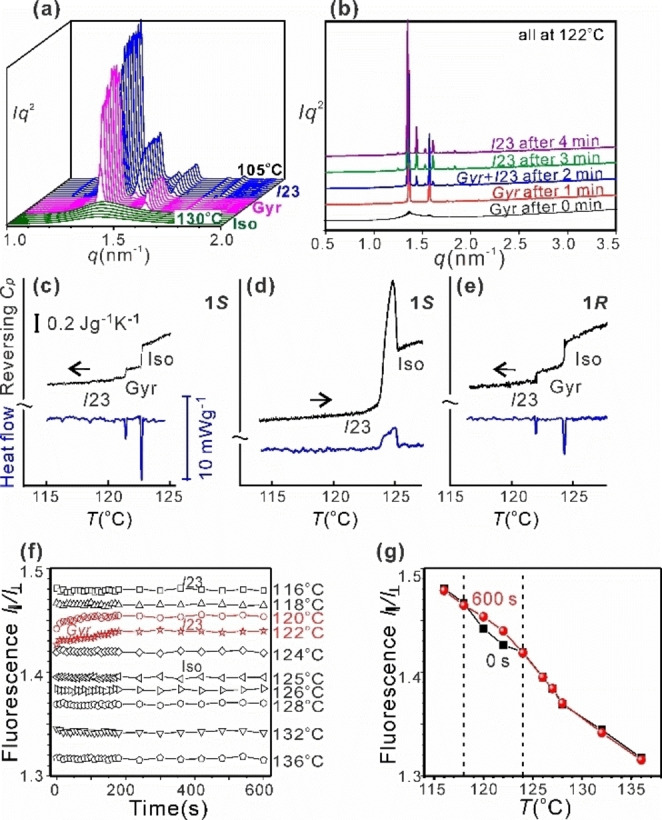
(a, b) Low‐angle XRD traces recorded (a) during 2 K/min cooling of **1*S*** from Iso through gyroid to *I*23, and (b) isothermally at 121 °C after cooling from Iso. (c–e) MDSC scans showing reversing heat capacity (black) and total heat flow (blue) through the LC–Iso transition region for (c) **1*S*** (cooling), (d) **1*S*** (heating) and (e) **1*R*** (cooling). Heat/cool rate 0.04 K/min, amplitude 0.07 K, period 20 s. (f,g) Ratio (*I*_//_ */I*_⊥_) of intensities of fluorescence of **1*S*** passing through analyser parallel and perpendicular to excitation polarization (f) as a function of annealing time at indicated temperatures following cooling from Iso. (g) *I*_//_ */I*_⊥_ values at the beginning of annealing (black) and 10 min later (red) vs. annealing temperature. *I*_//_ */I*_⊥_ reflects the system's rigidity.

No exotherm associated with the gyroid‐*I*23 transition could be seen by conventional scanning calorimetry (DSC, Figures S1). However ultraslow scanning by modulated DSC (MDSC, Figures 2c, e) shows two sharp exotherms in heat flow and two associated drops in reversing heat capacity (*c_p_*), the higher‐*T* and the lower‐*T* one corresponding to the Iso‐gyroid and gyroid‐*I*23 transitions. The gyroid is seen to have significantly higher *c_p_* than the *I*23. The isotropization temperature *T_i_* of the *I*23 phase on heating is 125 °C (Figures 2d and S2). However, the gyroid, while always metastable, when caught before its disappearance, melts on heating at 124 °C, only 1 K below the *I*23 (Figure S3).

As the compounds are fluorescent, their mobility was monitored as a function of *T* and time by measuring fluorescence depolarization. The sample was illuminated with linearly polarized light of 450 nm while emission was monitored with the analyser alternatively parallel and perpendicular to the polarizer during isothermal runs at different temperatures after a rapid cool from Iso. The ratio of intensities *I*_//_ */I* 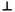 is plotted vs. time for **1*S*** in Figure 2f. *I*_//_ */I* 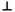 is a measure of rigidity of the system; a higher ratio means fewer molecules rotating away between excitation and emission. As seen in Figure 2f, while overall rigidity is the higher the lower the *T*, in general there is no change with annealing time. The two exceptions are at 120 and 122 °C where *I*_//_ */I* 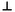 is initially slightly lower, rising to a steady value at roughly the time of the gyroid‐*I*23 transition shown by XRD. Figure 2g charts the *T‐*dependence of *I*_//_ */I* 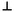 measured at the beginning (black) and end of the 10 min annealing period (red). The *T*‐range where the two curves depart, delimited by the vertical lines, is the range of formation of the metastable gyroid. We see that the gyroid is slightly more mobile than the *I*23 phase.

Analogous time‐ and *T*‐resolved experiments were performed next by circular dichroism spectroscopy, using synchrotron light. CD spectra of **1*R*** and **1*S*** at selected temperatures are shown in Figure 3a. Ellipticity at 355 nm, recorded during a cooling and heating run at 2 K/min, is plotted vs. *T* in Figure 3b for both enantiomers. The heating and cooling curves again depart in the *T*‐range where the gyroid forms on cooling. Most significantly, this shows that CD of the gyroid, while being slightly lower than that of the *I*23, is certainly *not zero or near zero*, as would be expected if the two networks were counter‐twisting. In other words and unexpectedly, this gyroid phase is *chiral*. Henceforth we will refer to it as the chiral gyroid, abbreviated cGyr. Also significant is the large pretransitional increase in CD over a ∼15 K interval in the Iso above the cubic phases, a subject to be dealt with in a separate future report.

**Figure 3:**
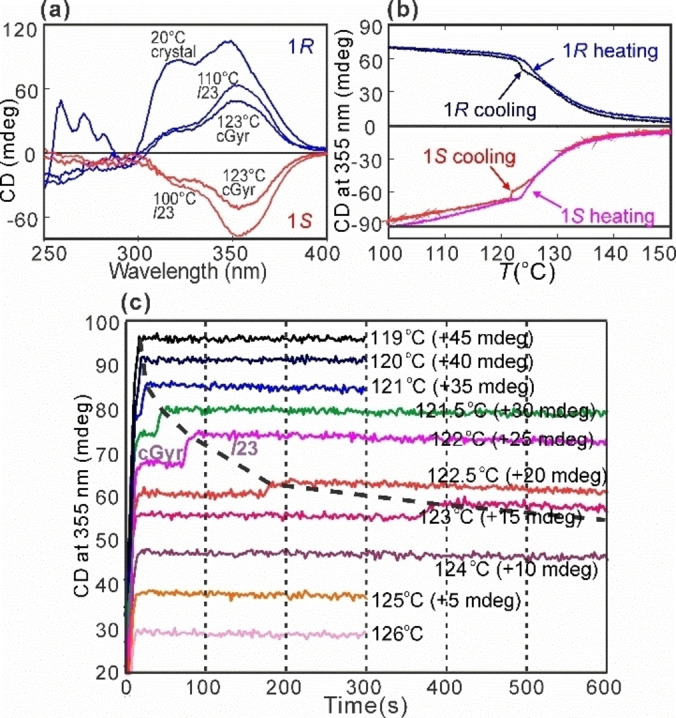
(a) CD spectra of films of **1*R*** (blue) and **1*S*** (red) at different temperatures. (b) CD ellipticity at 355 nm vs. *T* on heating and cooling at 2 K/min for **1*R*** and **1*S***. (c) CD at 355 nm vs. time of isothermal annealing at stated temperatures. For clarity, traces were shifted vertically by the amounts indicated. The step‐ups due to gyroid‐*I*23 transition are connected with a dashed line.

To ascertain conclusively that it is the gyroid that is chiral and not the *I*23 or the Iso, CD at 355 nm was monitored during isothermal runs at a series of temperatures following a rapid cool from 130 °C (Iso). The time evolution plots in Figure 3c show a small but distinct sharp step‐up in CD after an initial period whose length increases with *T*. This happens between 120 and 123 (±1) °C, precisely in the range where gyroid forms and disappears. Moreover, the plots show that the gyroid lifetime lengthens significantly as its melting point of 124 °C is approached.

Our explanation of the above results is as follows. The new transient chiral mesophase has all the XRD hallmarks of the gyroid, except for two weak Bragg reflections, (110) and (310), forbidden by the $Ia\bar{3}d$ spacegroup (Figure 4a and Table S3). The mirror symmetry is thus broken. Strictly, the spacegroup now is *I*4_1_32 (Q211), the same symmetry as found in structures of colorful butterfly wings.^[36]^ The structure can be described as “alternating gyroid”, which is chiral.[^37^, ^38^] However, in contrast to other cases of alternating gyroid,[^34^, ^35^, ^39^, ^40^] in the present cGyr phase both networks contain the same chemical species, albeit of different chirality. The almost negligible difference between diffraction intensities of the $Ia\bar{3}d$ and cGyr implies that the trajectories of the networks and the location of junctions are essentially the same. Packing optimization still requires that molecules at cores of the junctions are normal to the plane of the junction. As adjacent junctions are related by a 70.5° torsion (Figure 4f), normally the average twist angle between successive rafts would be *Φ*=70.5/*n_raft_*, where *n_raft_* is the number of rafts in an inter‐junction segment. As seen in Table S4, for compounds **1*R*** and **1*S*** *Φ*=8.0° in both the gyroid and the *I*23.

**Figure 4:**
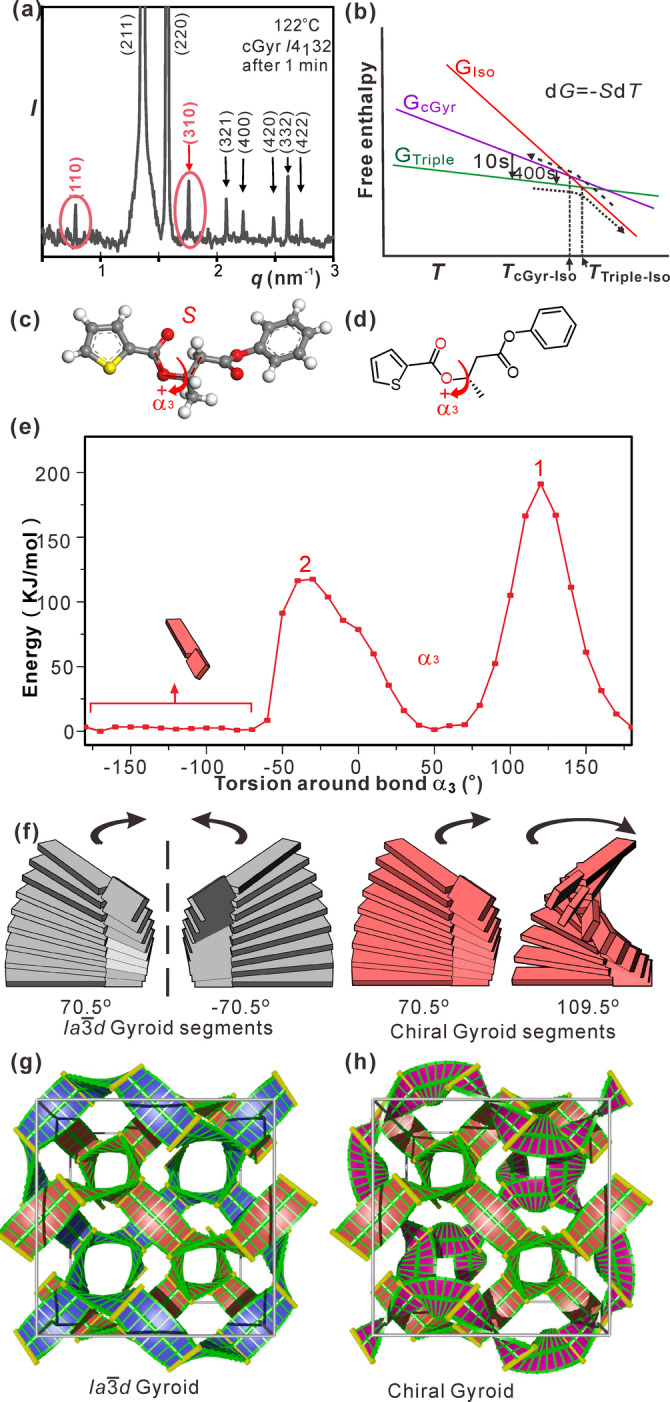
(a) Low‐angle X‐ray diffractogram of the gyroid phase in **1*S*** recorded 1 min after cooling from Iso to 122 °C. (b) Schematic *T*‐dependence of free energies of the Iso, *I*23 and gyroid phases near *T_i_*. Curved arrows show pathways on heating and cooling. (c, d) A segment of the **1*S*** molecule for which the energy was calculated as a function of torsion angle α_3_, displayed in (e) (adapted from Ref. [26]). (f) Helical stacking of rafts in an inter‐junction segment of each of the two networks, each raft represented by one “molecule”; left (grey): normal gyroid; right (red): chiral gyroid. The rightmost segment is “supertwisted” by 110.5°, others by 70.5°. (g, h) Models of a gyroid unit cell using spine‐and‐ribs ribbons, each rib representing a raft with molecules parallel to rib. (g) Achiral and (h) chiral gyroid. The “supertwisted” net in (h) is shown in purple. See also the Supporting Video.

The ability of a molecule to fit in a close‐packed chiral environment depends on whether its low‐energy conformations allow it. The main shape‐determining bond of a molecule of **1** is the C−O single bond of the butanoate, marked in Figures 4c, d with a curved arrow. There are two high energy barriers to rotation—see Figure 4e. The higher one is where the adjacent carbonyl oxygen clashes with the CH_3_ hydrogens, and the lower one is where it clashes with the nearby CH_2_ hydrogens. For **1*S*** the minimum between them, i.e. for α_3_ between −180° and −60°, symbolized by the red twisted board in Figure 4e, is far wider than that centred at +50°. Hence the former conformation is favoured. The opposite is true for **1*R***. In comparison, for an achiral molecule the two minima are just mirror images of each other.^[26]^

Figure 4f (left) shows schematically the packing of twisted board‐like cores of achiral molecules in a segment of each of the two networks of the achiral gyroid. The boards are drawn with their optimal twist angle α_3_ for best fit to each network, one having a positive and the other a negative sign. The total twist of the segment between junctions is ±70.5°. However, since in the chiral **1*S*** a positive α_3_ is less favoured than a negative one, and because conformational flexibility is allowed within the broad minimum in the negative α_3_ range, the molecules choose a negative intramolecular (α_3_) twist, and a left‐handed inter‐raft (column) twist *in both networks* (Figure 4f right). This means a higher column twist in the second network, making a total rotation of 180−70.5=109.5° between junctions, or a “supertwist” of 12.4° between rafts (Table S4). Unit cells of the gyroids in an achiral and a chiral compound, viewed along [100], are shown in Figures 4g, h, using ribbon representation. The Supporting Video shows a 3D model, first of a normal achiral gyroid with the left‐twisted (pink) and right‐twisted (blue) network. The blue net is then replaced, in two steps, by the “supertwisted” left‐handed net, painted red. The G minimum surface, itself achiral, is colored blue and red on opposite sides, indicating the chirality of the half‐spaces that they delimit. Note that, despite the blue network flipping to red, the half‐space in which it is confined remains blue, i.e. right‐handed.

The fact that *T_i_* of the “supertwisted” chiral gyroid is only 1 K lower than *T_i_* of the stable *I*23 phase, and yet that its lifetime shortens drastically with decreasing temperature, can be understood by considering the schematic *T*‐dependence of free energies *G*(*T*) in Figure 4b. The observed behavior is consistent with the downward slope of *G_cGyr_* (*T*) being steeper than that of *G*_*I*23_(*T*). Since *dG/dT*=−*S*, it follows that the gyroid has a higher entropy *S* than the *I*23, and becomes almost stable at *T_i_* of *I*23. As indicated by the vertical arrows symbolising cGyr‐*I*23 transition, its lifetime shortens at lower *T* because of increasing driving force for the transition, *G_cGyr_*−*G*_*I*23_=(*S_cGyr_*−*S*_*I*23_)(*T*_*I2*3‐*Iso*_−*T*). Compared with the *I*23, the higher entropy, hence higher disorder, of the cGyr is also consistent with its higher *c_p_* and higher mobility (Figures 2c, e, f). That the gyroid is a high‐entropy phase is also borne out by our preliminary experiments on a number of achiral polycatenar systems where it often appears as the high‐*T* cubic, either stable or metastable. In general, it is also found to be more tolerant to defects and, unlike *I*23, its XRD pattern sometimes signals very high dynamic lattice distortion. Remarkably, as shown here, it is even able to tolerate the “wrong” twist of one of its networks. We note that at *T*_*Iso‐cGyr*_ the Iso liquid is already highly chiral (Figure 3), seemingly containing short isochiral twisted network fragments. Isochirality of the two networks in cGyr thus ensures a smooth sequence Iso→cGyr→*I*23 without the need for their rewind.

Preliminary experiments on the racemic mixture of **1*S*** and **1*R*** have indicated that there a metastable gyroid also forms first.

Based on these findings we conclude that the omnipresence of the gyroid is at least partly due to its high adaptability and tolerance to defects, including variation in chemical structure. Thus it can even accommodate a strongly chiral mesogen, inverting and “supertwisting” one of its two networks and becoming a chiral phase. In view of its current and future applications, including in photonics, in separation membranes and as cubosomes for delivery of chiral drugs, understanding its response to chirality is of considerable interest.

## Supporting Information

Supporting Information, including a supporting video, is available from the Wiley Online Library or from the author.

## Conflict of interests

The authors declare no conflict of interest.

## Supporting information

As a service to our authors and readers, this journal provides supporting information supplied by the authors. Such materials are peer reviewed and may be re‐organized for online delivery, but are not copy‐edited or typeset. Technical support issues arising from supporting information (other than missing files) should be addressed to the authors.

Supporting Information

Supporting Information

## Data Availability

The data that support the findings of this study are available in the supplementary material of this article.
