# Supplementary material for: Supertwisted Chiral Gyroid Mesophase in Chiral Rod‐Like Compounds
Source: Angew Chem Int Ed Engl. 2024 May 2;63(23):e202403156. doi: 10.1002/anie.202403156 (PMC11497307; doi:10.1002/anie.202403156)
Supplement: Supplementary file 1 — Supporting Information [file ANIE-63-e202403156-s002.pdf]

Angewandte Chemie
Eine Zeitschrift der Gesellschaft Deutscher Chemiker

Supporting Information

Supertwisted Chiral Gyroid Mesophase in Chiral Rod-Like Compounds

Y. Wang, S.-G. Yang, Y.-X. Li, Y. Cao, F. Liu, X.-B. Zeng, L. Cseh*, G. Ungar**

Supporting Infomation

Supertwisted Chiral Gyroid Mesophase in Chiral Rod-like Compounds

Yan Wang[1], Shu-Gui Yang[1], Ya-Xin Li[2], Yu Cao,[1] Feng Liu[1], Xiang-Bing Zeng[3,*], Liliana Cseh[4,*] and Goran Ungar[1,3,*]

1. Shaanxi International Research Centre for Soft Matter, State Key Laboratory for Mechanical Behaviour of Materials, Xi'an Jiaotong University, Xi'an, 710049, China.
2. School of Chemistry and Chemical Engineering, Henan University of Technology, Zhengzhou 450001, China.
3. Department of Materials Science and Engineering, University of Sheffield, Sheffield S1 3JD, UK.
4. Romanian Academy, Coriolan Dragulescu Institute of Chemistry, Timisoara 300223, Romania

Contents

S1. Materials and methods.......... 2
S2. Additional DSC data and thermograms.......... 3
S3. Additional X-ray data.......... 4
S4. Calculation of inter-raft twist and number of molecules per raft.......... 7
S5. Synthetic Procedures and Characterization Data.......... 8
    S5.1 Synthesis of compounds N*S and* N*R*.......... 9
    S5.2 Synthesis of compound 1*S*.......... 11
    S5.3 Synthesis of 1*R* compound.......... 18
S6. References.......... 22

S1. Materials and methods

Synthesis. All chemicals and commercially available reagents were used as purchased without further purification. Dry solvents were taken from a solvent purification system. Glassware used for water-free reactions was dried for 24 h at 120 °C before use. All reactions were performed under an atmosphere of nitrogen unless stated otherwise. Columns were packed with silica gel (300–400 mesh) as the stationary phase.

Chemical analysis. ^1H and ^{13}C NMR and 2D NMR spectra were recorded on a Brüker DMX-600 (^1H: 600 MHz; ^{13}C: 150 MHz) spectrometer at 298 K. The ^1H and ^{13}C NMR chemical shifts are reported relative to residual solvent signals. Coupling constants (J) are denoted in Hz and chemical shifts (δ) in ppm. Multiplicities are denoted as follows: br = broad, s = singlet, d = doublet, t = triplet, m = multiplet. High-resolution mass spectral (HRMS) data were obtained on an electrospray (ESI) mass time-of-flight spectrometer (Waters i-Class VION IMS QToF). Elemental analysis was done on Elementar Vario EL cube/Vario OXY cube element analyzer.

Conventional differential scanning calorimetry (DSC) thermograms were recorded on a TA DSC250 calorimeter purged with nitrogen and using a RCS90 two-stage refrigeration unit. Heating and cooling rate was 5 K/min unless stated otherwise. Temperature, thermal resistance of the cell and enthalpy calibration were performed using Indium and distilled water, with linear interpolation of error. Peak transition temperatures are quoted, corrected for thermal lag. Temperature calibration for cooling runs was performed by linear extrapolation of temperature error to negative heating rates.

The same instrument was used for **modulated DSC (MDSC)** for near-equilibrium measurement of heat capacity. The linear cooling/heating rate was 0.04 K/min, the amplitude of superimposed temperature oscilation was 0.07 K and the period of oscillation was 20 s.

For **polarized optical microscopy** (POM) an Olympus BX51 microscope was used equipped with a Linkam LTS420E hot stage and a T95-HS controller. The samples were heated to the isotropic melt between glass slides and then cooled to desired temperature at varying rates, as required.

For **depolarized fluorescence microscopy** the same BX-51 microscope was employed. For this purpose reflection geometry was used, with a PE-300 white light source (CoolLED), a BP 460-490 excitation filter, a DM 500 dichroic mirror, and a LP 520 emission filter. The excitation light with a wavelength around 450 nm was passed through a polarizer before reaching the sample. The fluorescence was recorded after passing through an analyzer that was parallel ($I_{\parallel}$) or perpendicular ($I_{\perp}$) to the polarization direction of exciting light, respectively. The samples were heated above isotropic temperature (150 °C) and then cooled to preset temperature (114 –134 °C) at a rate of 30 K/min. Fluorescence anisotropy was defined as the ratio between the parallel ($I_{\parallel}$) and perpendicular ($I_{\perp}$) fluorescence intensities.

Chiro-optical measurements were carried out at Beamline B23 of Diamond Light Source using the Mueller matrix method[S1]which is capable of separating the effects of linear birefringence and dichroism from those of CD and optical

activity. In this work this separation was relevant only for the crystal phase, as all other phases studied (Iso, cGyr, *I*23) are optically isotropic. The synchrotron-generated light beam was reflected vertically which allowed it to pass through the sample film between quartz glass windows lying horizontally to avoid flow of the liquid. The sample sandwich was held in a Linkam hot-stage.

Synchrotron X-ray diffraction. Small/wide-angle powder X-ray scattering (SAXS/WAXS) experiments were conducted at beamline BL16B1 of Shanghai Synchrotron Radiation Facility, China, and beamline I22 of Diamond Light Source, U.K. Two Pilatus 2M detectors (Dectris) were used simultaneously at Diamond, one for SAXS and another for WAXS. X-ray energy was 12.4 keV. At both facilities the samples in 1mm glass capillaries were held in a modified Linkam hot stage. During the experiment the capillary was rotated by an electromotor at ca 200 rpm to improve signal averaging and reduce radiation damage. Separate **off-line SAXS/WAXS** experiments were carried out on a SAXSpoint 2.0 instrument from Anton Paar, equipped with a 50 kV/1 mA sealed-tube microfocus generator, multilayer focusing mirrors and an Eiger R 1M decector (Dectris). Powder samples were held in 1mm glass capillaries in an Anton Paar Heated Sampler 2.0, all in vacuum. Sample temperature was calibrated by several standard substances. Sample-to-detector distance, in detector pixel units, was calibrated by silver behenate. Fibrefix software was used for general data processing.

S2. Additional DSC data and thermograms

Table S1. Transition temperatures of compound **1*R*** and **1*S*.**

Compd	T/°C [$\triangle H$/J g^{-1}]^a
	1st heat: Cr 87 [16.3] *I*23 125 [2.06] Iso
**1*R***	Cool: Iso 124^b [0.80] cGyr 121.5^b *I*23 43 [4.65] Cr
	2nd heat: Cr 53 [4.5] *I*23 125 [2.06] Iso
	1st heat: Cr 88 [14.0] *I*23 125 [1.95] Iso
1S	Cool: Iso 123^b [0.80] cGyr 121.7^b *I*23 42 [4.3] Cr
	2nd heat: Cr 53 [4.3] *I*23 125.04 [1.95] Iso

^{a}Peak DSC transition temperatures and enthalpies at 5 K·min^{-1}.

^{b}From MDSC at 0.04 K/min.

Cr = crystal, cGyr = chiral gyroid cubic phase with $I4_132$ symmetry; *I*23 = chiral triple network cubic phase with *I*23 symmetry. Iso = isotropic melt. For DSC thermograms see Fig. S1.

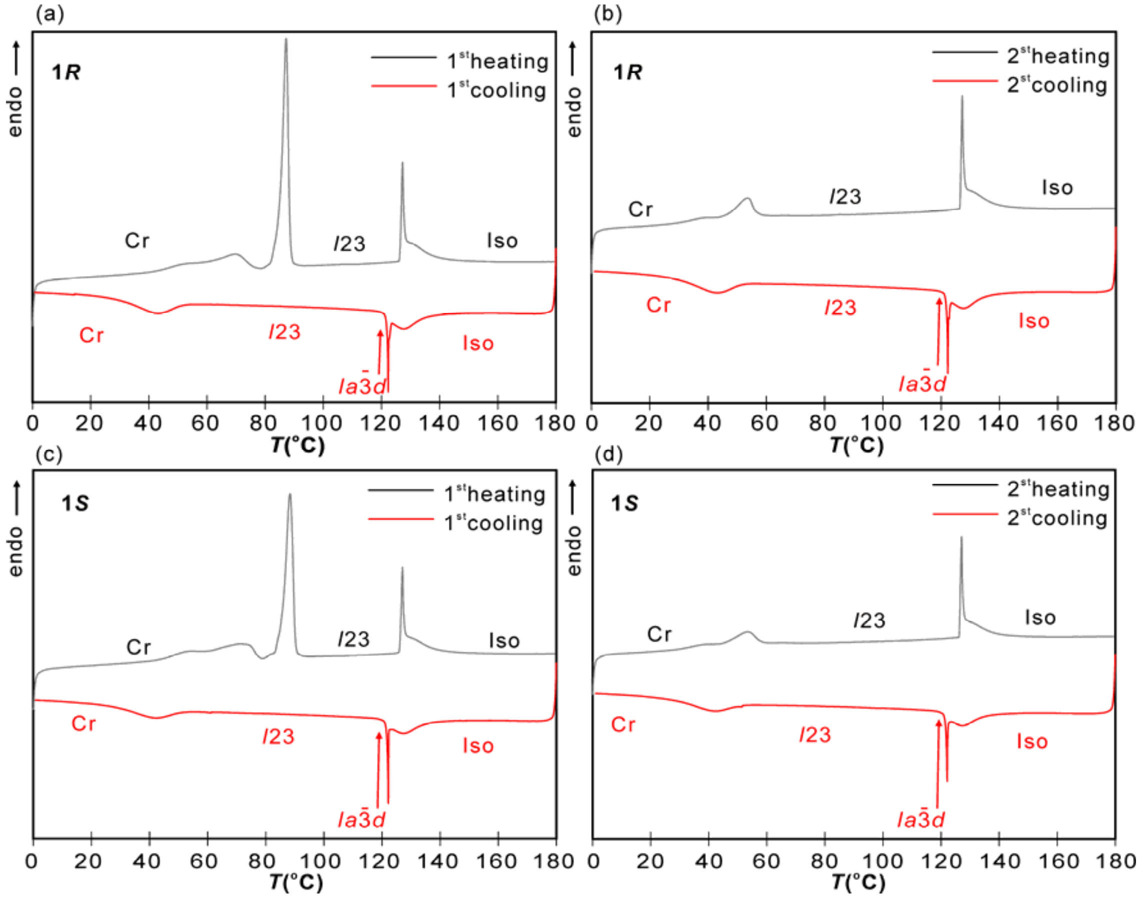

Figure S1. (a,c) First heating and cooling traces of compounds (a) **1*R*** and (c) **1*S***. (b,d) Second heating and cooling traces of compounds (b) **1*R*** and (d) **1*S***. All scan rates 5 K min^{-1}.

S3. Additional X-ray data

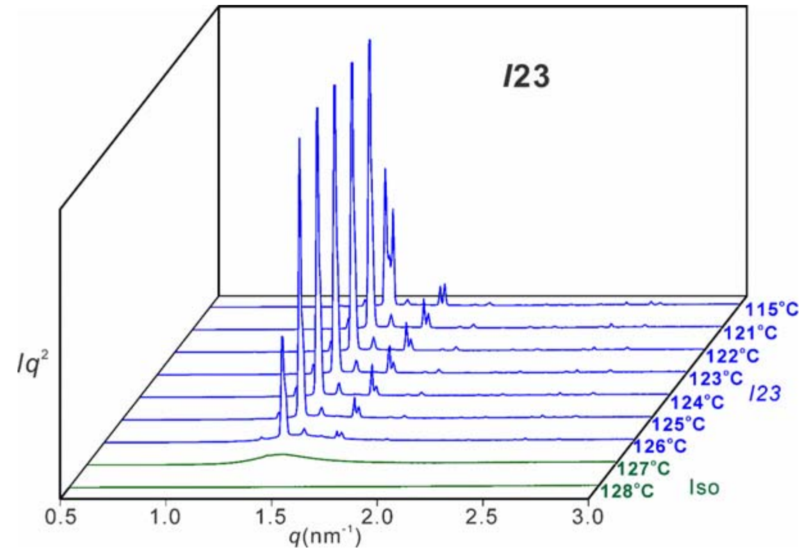

Figure S2. SAXS diffractogram of **1*S*** on heatting from *I*23 to Iso at 2 K/min.

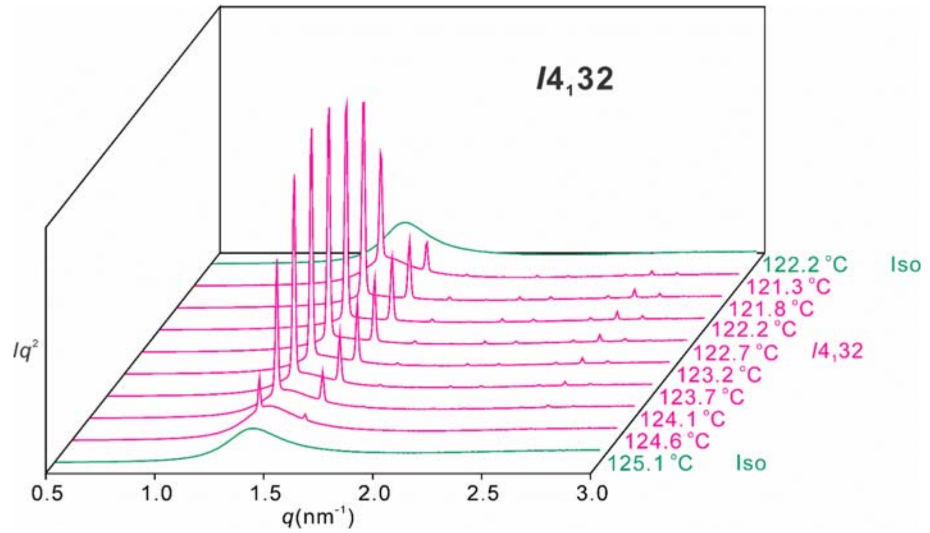

Figure S3. SAXS diffractograms of **1*S*** showing, from back to front, the Iso phase on rapid cooling (30 K/min), the immediate formation of the cGyr $I4_132$ phase, followed by heating at 2 K/min. The cGyr phase persists to 124°C.

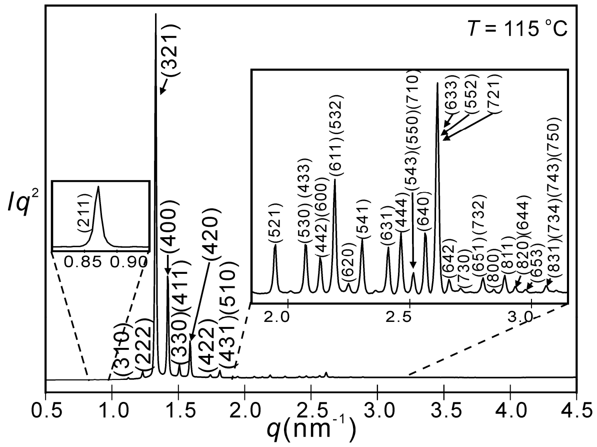

Figure S4. SAXS diffractogram of **1*S*** at 115°C on heating. $I23$ phase. Intensity scale is expanded in the inset.

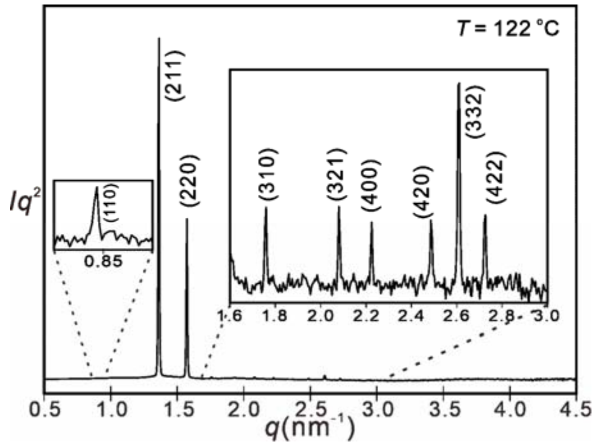

Figure S5. SAXS diffractogram of **1*S*** at 122°C on cooling after annealing for 1 min. $\mathbf{I4_132}$ chiral gyroid phase. Intensity scale is expanded in the inset.

Table S2: Indices, experimental and calculated *d*-spacings, parameters of the cubic phase with ***I*23** symmetry in **1*S*** recorded at 105 °C in the cooling process with cooling rate 2 °C/min.

(*hkl*)	$d_{obs.}$- spacings (nm)	$d_{cal.}$- spacings (nm)
(211)	7.21	7.20
(310)	5.58	5.58
(222)	5.09	5.09
(321)	4.71	4.71
(400)	4.41	4.41
(411/330)	4.16	4.16
(420)	3.95	3.94
(422)	3.60	3.60
(431/510)	3.46	3.46
(521)	3.22	3.22
(530/433)	3.02	3.02
(600/442)	2.93	2.94
(611/532)	2.86	2.86
(541)	2.72	2.72
(631)	2.60	2.60
(444)	2.54	2.54
(710/550/543)	2.49	2.49
(640)	2.44	2.44
(721/552/633)	2.40	2.40
a_{cub} = 17.63 nm		

Table S3: Miller indices, experimental and calculated *d*-spacings, parameters of the cubic phase with ***I*4$_1$32** symmetry in **1*S*** recorded at 122 °C after annealing for 1 min.

(*hkl*)	$d_{obs.}$- spacings (nm)	$d_{cal.}$- spacings (nm)
(110)	8.01	8.00
(211)	4.62	4.62
(220)	4.00	4.00
(310)	3.57	3.58
(321)	3.02	3.02
(400)	2.82	2.83
(420)	2.53	2.53
(332)	2.41	2.41
(422)	2.31	2.31
a_{cub} = 11.31 nm		

S4. Calculation of inter-raft twist and number of molecules per raft

Table S4: Calculation of number of molecules per raft and twist angle between rafts in **1*S*** (a raft is a single-molecule-thick section of a segment).

Phase	*Lattice parameters* (nm)	V_{cell} (nm^{3}*10^{2})^{a}	V_{mol} (nm^{3})^{b}	$\mu_{cell}{}^{c}$	$\mu_{raft}{}^{d}$	Φ (°)^{e}
***I*4$_1$32**	11.31	53.04	2.30	626	3.0	8.0, 12.4
***I*23**	17.63	14.43		2302	2.9	8.0

a: The volume of the unit cell: $V_{cell}=a^3$

b: The volume of the molecule is calculated by the density method, $V_{mol} = M/\rho N_A$, where ρ is density, *M* is molecular mass and N_A is Avogadro's number. Based on data on similar materials, ρ = 0.95 g cm^{-3} is assumed.

c: Number of molecules per unit cell μ_{cell} is calculated from $\mu_{cell} = V_{cell}/V_{mol}$

d: Number of molecules per raft μ_{raft}: In ***I*4$_1$32** phase there are a total of 24 inter-junction network segments in the unit cell. The length of each segment is $(\sqrt{2}/4)a_{cub} = 0.354a_{cub}$. The number of molecules in a segment is $\mu_{cell}/24$. the number of rafts Assuming the thickness of a raft, as usual in such molecules and based on WAXS, to be 0.45 nm, we get the number of rafts

per segment as n_{raft} =$0.354a_{cub}$/0.45 and the number of molecules per raft as μ_{raft} = μ_{cell}/(24×n_{raft})=3.0. In ***I*23** phase the total length of all three networks in a unit cell is 20.68a_{cub} and μ_{raft} is calculated as μ_{raft} = μ_{cell}/n_{raft} = μ_{cell}/(20.68a_{cub}/0.45).

e: Twist angle Φ between successive rafts: The total twist angle of a segment between two junctions in a $I4_132$ cell is either 70.5° or 109.5° for the "twisted" and the "supertwisted" network, respectively. These angles multiplied by 24/n_{raft} then give Φ. In ***I*23** phase the segment length of the inner and outer networks is 0.29a_{cub} = 5.11 nm, containing 11.4 rafts. The twist angle of each segment between junctions is 90°, giving Φ = 8.0°. In the middle networks, the length of the shorter and longer segments is 0.145a_{cub} and 0.355a_{cub}, respectively. The twist angles of the shorter and longer segments are 44.6° and 109.3°, respectively. The twist angle between rafts in this network is thus also Φ = 8.0°.

S5. Synthetic Procedures and Characterization Data

Abbreviations:

MeOH - methanol

THF- tetrahydrofuran

PE – petroleum ether

DCM - dichloromethane

AcOH - acetic acid

DMAP - 4-dimethylaminopyridine

EDC·HCl - 1-ethyl-3-(3-dimethylaminopropyl)carbodiimide hydrochloride

r.t . - room temperature

h – hour

aq - aqueous

The **A-G** and **L** compounds were synthesized according to reported literature.[S2-S5]

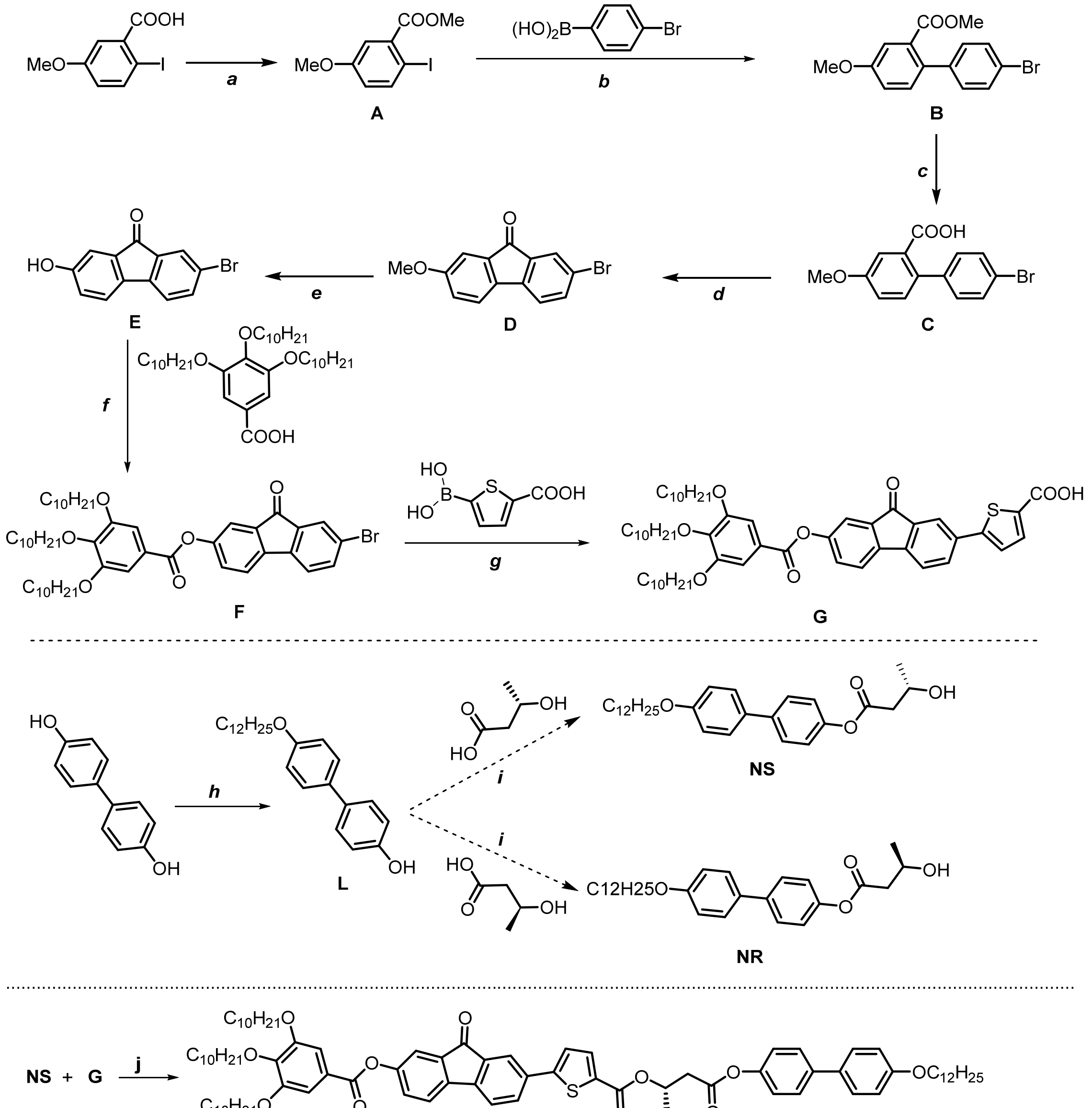

Scheme S1. Synthesis of **1*R*** and **1*S*** compounds. Reagents and conditions: (a) H_2SO_4, MeOH, 80 °C; (b) (g) $Pd(PPh_3)_2Cl_2$, THF, 2M Na_2CO_3. 80 °C; (c) THF, MeOH, KOH, 80 °C; (d) $SOCl_2$, $AlCl_3$, DCM, r.t.; (e) HBr 48%, AcOH, 120 °C; (f)(i) EDC·HCl, DMAP, DCM, r.t.; (h) $C_{12}H_{25}Br$, K_2CO_3, KI, DMF, H_2O; (j) (I) $SOCl_2$, reflux; (II) pyridine, DCM, r.t.

S5.1 Synthesis of compounds N*S and* N*R*

The 4'-(dodecyloxy)-[1,1'-biphenyl]-4-ol (**L**) (1.1 g, 3 mmol) and (*R*)-3-

hydroxybutanoic acid or (*S*)-3-hydroxybutanoic acid (312.3 mg, 3 mmol) was placed in 50 ml round bottom flask and DCM (15 ml) was added. Then EDC·HCl (863 mg, 4.5 mmol) and DMAP (147 mg, 1.2 mmol) were added at 0 °C and stirred for 20 h at r.t. The resulted mixture was diluted with extra DCM solvent (20 ml), and then successively washed with H_2O (3×20 ml), 10% NaOH (10 ml) and H_2O (2×15 ml). The organic phase was separated, dried over anhydrous Na_2SO_4, filtered and concentrated. The crude product was purified by column chromatography to afford the pure product:

N*R* as a white solid (1.06 g, 80 %). (DCM/PE, 10:1). **^{1}H NMR** (600 MHz, $CDCl_3$) δ 7.54 (d, *J* = 8.5 Hz, 2H), 7.48 (d, *J* = 8.6 Hz, 2H), 7.13 (d, *J* = 8.5 Hz, 2H), 6.96 (d, *J* = 8.6 Hz, 2H), 4.40 – 4.28 (m, 1H), 3.99 (t, *J* = 6.5 Hz, 2H), 2.83 – 2.65 (m, 2H), 1.91 – 1.71 (m, 2H), 1.53 – 1.40 (m, 2H), 1.34 (d, 3H), 1.31 – 1.23 (overlap, 16H), 0.88 (t, *J* = 6.9 Hz, 3H)

^{13}C NMR (150 MHz, $CDCl_3$) δ 171.6, 158.8, 149.2, 139.0, 132.6, 128.1, 127.8, 121.7, 114.8, 68.1, 64.3, 42.9, 31.9, 29.68, 29.66, 29.63, 29.61, 29.43, 29.37, 29.30, 26.1, 22.7, 22.5, 14.1. **ESI-HR-MS**: *m/z* calcd. for $C_{28}H_{41}O_4^+$, ([M]+H$^+$). 441.2999, found 441.2994.

N*S* as a white solid (1.10 g, 83 %). (DCM/PE, 10:1). **^{1}H NMR** (600 MHz, $CDCl_3$) δ 7.54 (d, *J* = 8.5 Hz, 2H), 7.48 (d, *J* = 8.6 Hz, 2H), 7.13 (d, *J* = 8.5 Hz, 2H), 6.96 (d, *J* = 8.6 Hz, 2H), 4.40 – 4.28 (m, 1H), 3.99 (t, *J* = 6.5 Hz, 2H), 2.83 – 2.65 (m, 2H), 1.91 – 1.71 (m, 2H), 1.53 – 1.40 (m, 2H), 1.34(d, 3H), 1.32 – 1.20 (overlap, 16H), 0.88 (t, *J* = 6.9 Hz, 3H). **^{13}C NMR** (150 MHz, $CDCl_3$) δ 171.6, 158.8, 149.2, 139.0, 132.6, 128.1, 127.7, 121.7, 114.8, 68.1, 64.3, 43.0, 31.9, 29.68, 29.66, 29.63, 29.61, 29.43, 29.37, 29.30, 26.1, 22.7, 22.5, 14.1. **ESI-HR-MS**: *m/z* calcd. for $C_{28}H_{41}O_4^+$, ([M]+H$^+$). 441.2999, found 441.2994.

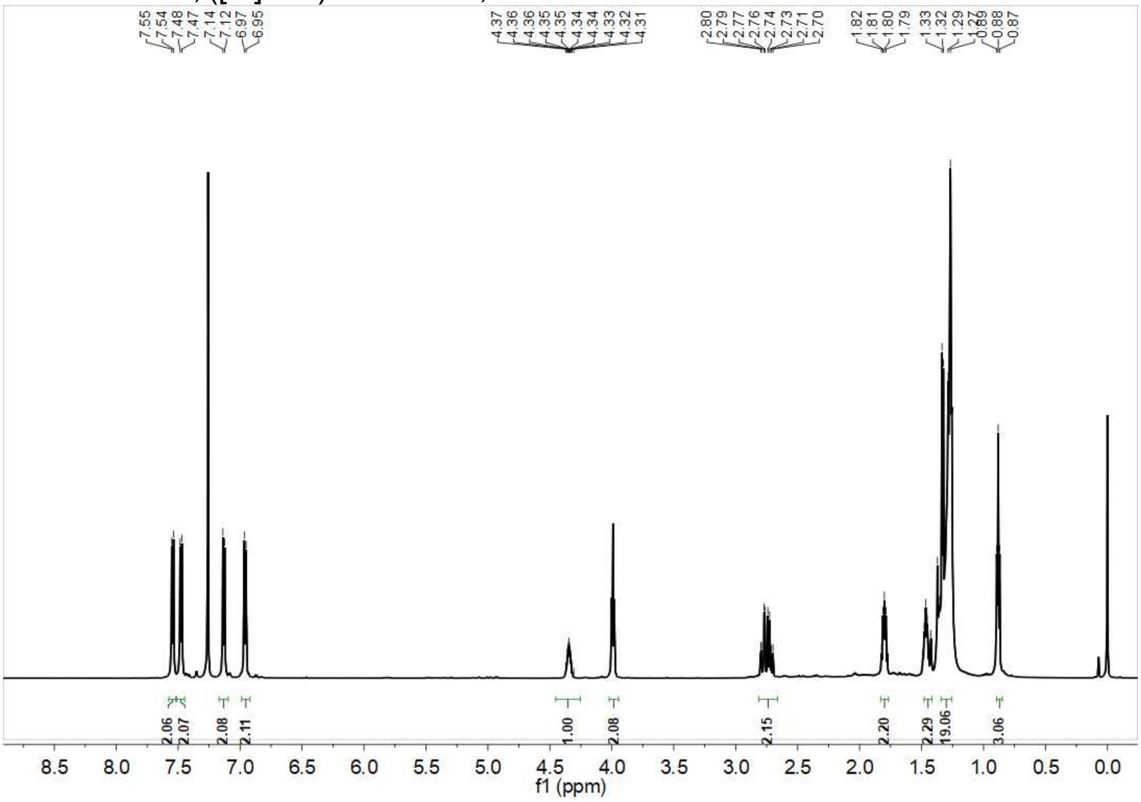

Figure S6. ^{1}H NMR spectrum (600 MHz, $CDCl_3$, 295 K) of **N*S***.

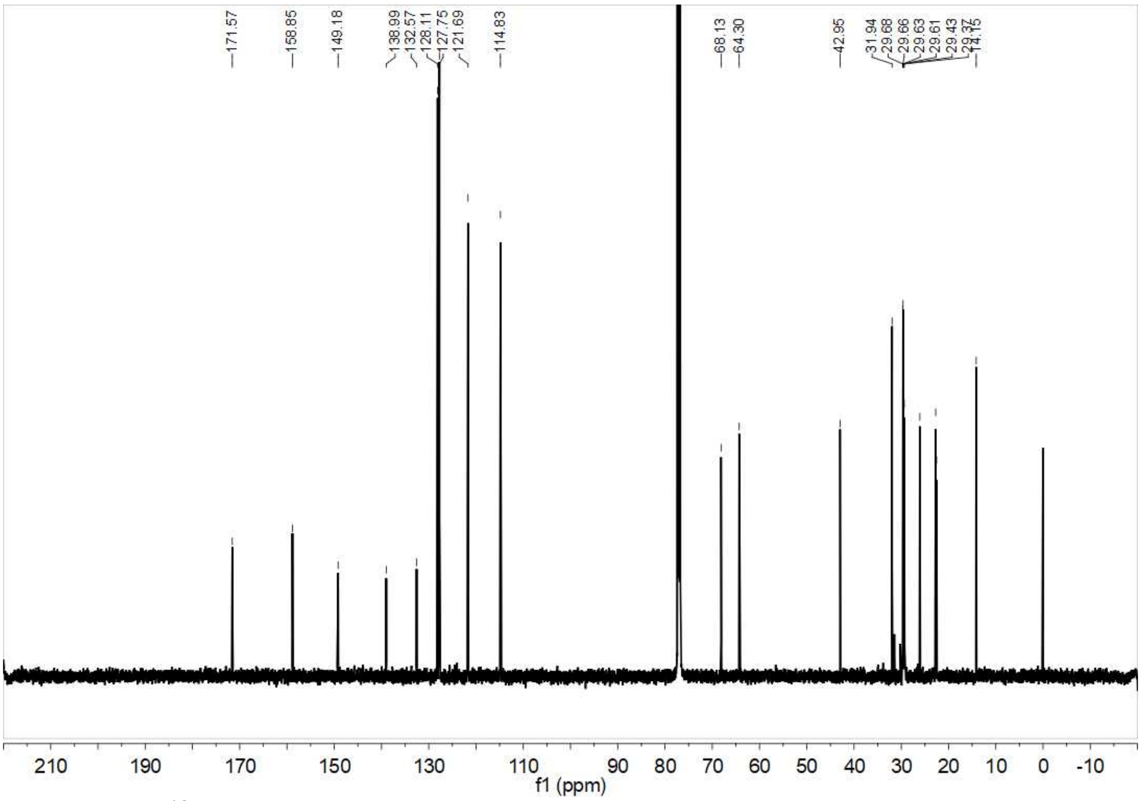

Figure S7. ^{13}C NMR spectrum (150 MHz, $CDCl_3$, 298 K) of **N*S***.

S5.2 Synthesis of compound 1*S*

The mixture of 5-(9-oxo-7-((3,4,5-tris(decyloxy)benzoyl)oxy)-9H-fluoren-2-yl) thiophene-2-carboxylic acid (**G)** (895 mg, 1 mmol) and $SOCl_2$ (5 ml) was refluxing for 30 min. The excess of $SOCl_2$ was removed under vacuum, and then dry pyridine (0.5 ml), DCM (5 ml) and 4'-(decyloxy)-[1,1'-biphenyl]-4-yl (*S*)-3-hydroxybutanoate (**N*S***) (440.29 mg, 1 mmol) were added. This mixture was stirred at r.t. overnight. The extra solvent DCM (50 ml) was added and the organic phase was separated. This was washed with saturated aq NaCl (2×20 ml), dried over anhydrous $MgSO_4$, concentrated under vacuum to afford the crude product. This was purified by column chromatography (DCM/PE = 10:1) to obtain ***S* compound** as a yellow solid (921.75 mg, 70 %). **^{1}H NMR** (600 MHz, $CDCl_3$) δ 7.94 (d, *J* = 1.5 Hz, 1H), 7.81 (d, *J* = 3.9 Hz, 1H), 7.77 (dd, *J* = 7.8, 1.7 Hz, 1H), 7.60 (d, *J* = 8.0 Hz, 1H), 7.56 (d, *J* = 7.8 Hz, 1H), 7.54 – 7.49 (m, 3H), 7.46 (dd, 2H), 7.40 (s, 2H), 7.38 – 7.34 (m, 2H), 7.12 (dd, 2H), 6.93 (dd, 2H), 5.65 (m, 1H), 4.06 (m, 6H), 3.98 (t, *J* = 6.6 Hz, 2H), 3.05 (dd, 1H), 2.92 (dd, 1H), 1.88 – 1.81 (m, 4H), 1.81 – 1.74 (m, 4H), 1.56 (d, *J* = 6.3 Hz, 3H), 1.54 – 1.43 (m, 8H), 1.40 – 1.21 (overlap, 52H), 0.88 (m, 12H). **^{13}C NMR** (150 MHz, $CDCl_3$) δ 192.0, 168.8, 164.8, 161.2, 158.8, 153.0, 152.2, 149.8, 149.3, 143.8, 143.3, 141.3, 138.9, 135.9, 135.3, 134.7, 134.3, 132.8, 132.6, 132.4, 128.1, 128.0, 127.7, 124.3, 123.3, 122.0, 121.7, 121.4, 121.0, 118.6, 114.8, 108.6, 73.7, 69.3, 68.5, 68.1, 41.1, 32.0, 31.9, 30.4, 29.8- 29.5, 29.4-29.2, 26.10, 26.07, 22.73, 22.71, 20.2, 14.1. **ESI-HR-MS**: *m/z* calcd. for $C_{83}H_{113}O_{11}S^+$, ([M]+H$^+$). 1317.7998, found 1317.80156. **EA**: calcd. for $C_{83}H_{112}O_{11}S$: C 75.65 %, H 8.57 %, S 2.43 %, found: C 75.63 %, H 8.56 %, S 2.47 %.

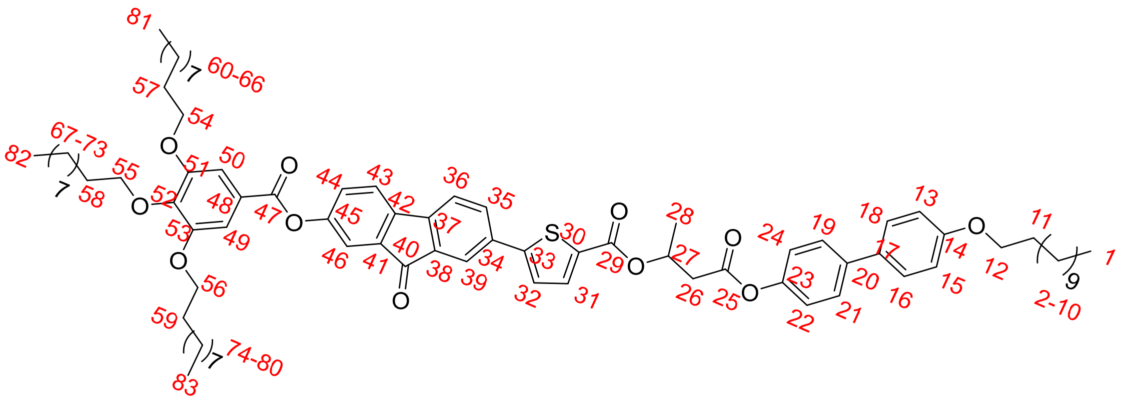

Table S5. Full NMR assignment of compound **1*S***

Position	1H-NMR	13C-NMR	H-H	HSQC	HMBC
1	0.88, 12H, (9H from pos. 81-83)	14.1		14.1	
2-9	1.21-1.40, 52H (36 H from pos. 61-66, 68-73 and 75-80).	22.7<br>32.0<br>29.2-29.8			
10	1.43-1.54, m, 2H	26.1			
11	1.74-1.81, m, 4H (2H from pos. 58)	30.4	1.79 – 3.98<br>H11 – H12	1.76	
12	3.98, t, 2H	68.1	3.98 – 1.79<br>H12 – H11	68.1	
13, 15	6.93, dd, 2H,<br>Jo = 8.7,<br>Jm = 1.9	114.8	6.93 – 7.46<br>H13,15– H16,18	114.8	H13+15<> C14 = 158.8<br>>C17 = 132.6<br>> C13+15 = 114.8
14	-	158.8			
16, 18	7.46, dd, 2H	128.1	7.46 – 6.93<br>H16,18 – H13,15	128.1	H16+18<> C14 = 158.8<br>>C20 = 138.9<br>> C16+18 = 128.1
17	-	132.6			

19, 21	7.51, m, 3H, (1H from pos. 46)	127.7	7.51 – 7.12<br>H19,21– H22,24	127.7	H19+21<> C23 = 149.3<br>>C17 = 132.6<br>>C19+21 = 127.7
20	-	138.9			
22, 24	7.12, dd, 2H,<br>Jo = 8.7,<br>Jm = 1.9	121.7	7.12 – 7.51<br>H22,24– H19,21	121.7	H22+24<> C23 = 149.3<br>>C20 = 138.9<br>> C22+24 = 121.7
23	-	149.3			
25	-	168.8			
26	3.05 + 2.92, 2H	41.1		41.1	H26-C25
27	5.65, m, 1H	68.5		68.5	
28	1.56, d, 3H,<br>J = 6.3	20.1		20.1	
29	-	161.2			
30	-	132.8			
31	7.81, d, 1H,<br>J = 3.9	134.7	7.81 – 7.37<br>H31- H32	134.7	H31<> C29 = 161.2<br>>C33 = 149.8<br>> C30 = 132.8<br>> C32 = 124.3
32	7.37, d, 1H	124.3	7.37 – 7.81<br>H32-H31	124.3	H32<> C33 = 149.8<br>>C17 = 132.6<br>> C319+21 = 127.7
33	-	149.8			
34	-	134.3			
35	7.77 dd, 1H,<br>Jo = 7.8,<br>Jm = 1.7	132.3	7.77 – 7.56<br>H35 - H36	132.3	H35<>C33 = 149.8<br>> C30 = 132.8<br>> C34 = 134.3
36	7.56, d, 1H,<br>J = 7.8	121.0	7.56 – 7.77<br>H36 – H35	121.0	H36<> C42 = 141.3<br>>C34 = 134.3
37	-	143.8			
38	-	135.3			
39	7.94, d, 1H,	122.0		122.0	H39<>C40 =192.0

	Jm = 1.5				>C33 = 149.8<br>>C37 = 143.8<br>> C35 = 132.3
40	-	192.0			
41	-	135.9			
42	-	141.3			
43	7.60, d, 1H,<br>Jo = 8.0	121.4	7.60 – 7.35<br>H43 – H44	121.4	H43<> C45 = 152.2<br>>C37 = 143.8<br>> C41 = 135.9
44	7.35, dd, 1H	128.0	7.35 - 7.60<br>H44 – H43	128.0	H44<> C42 = 141.3<br>>C46 = 118.6
45	-				
46	7.53, m, 3H, (2H from pos. 19 and 21)	118.6		118.6	H46<> C40 w* = 192.0<br>>C45 w* = 152.2<br>> C44 = 128.0<br>> C42 = 141.2
47	-	164.8			
48	-	123.3			
49, 50	7.40, s, 2H	108.6		108.6	H49 +50<> C47 = 164.8<br>>C51+53 = 153.0<br>>C52 = 143.3<br>> C48 = 123.3<br>>C49+50 = 108.6
51, 53	-	153,0			
52	-	143.3			
54, 56	4.05, m, 6H, (2H from pos. 55)	73.6	4.05 – 1.84<br>H54,56 - H57,59	73.6	
55	4.06, m, 6H, (4 H from pos. 54 and 56)	69.3	4.06 – 1.78<br>H55 – H58	69.3	
57, 59	1.84, m, 4H	29.7	1.84 – 4.05<br>H57,59 – H54,56		

58	1.78, m, 4H (2H from pos 11)	29.7	1.78 – 4.06<br>H58 – H55		
60, 74	1.49, m, 6H (2H from pos. 67)	26.1			
61-66<br>68-73<br>75-80	1.21-1.40 overlap, 52H, (16H from pos. 2-9)	22.7<br>29.3-29.6<br>31.9			
67	1.49, m, 6H, (4H from pos. 60 and 74)	26.1			
81-83	0.88, m, 12H, (3H from pos. 1)	14.1		14.1	

*w – the signal is weak

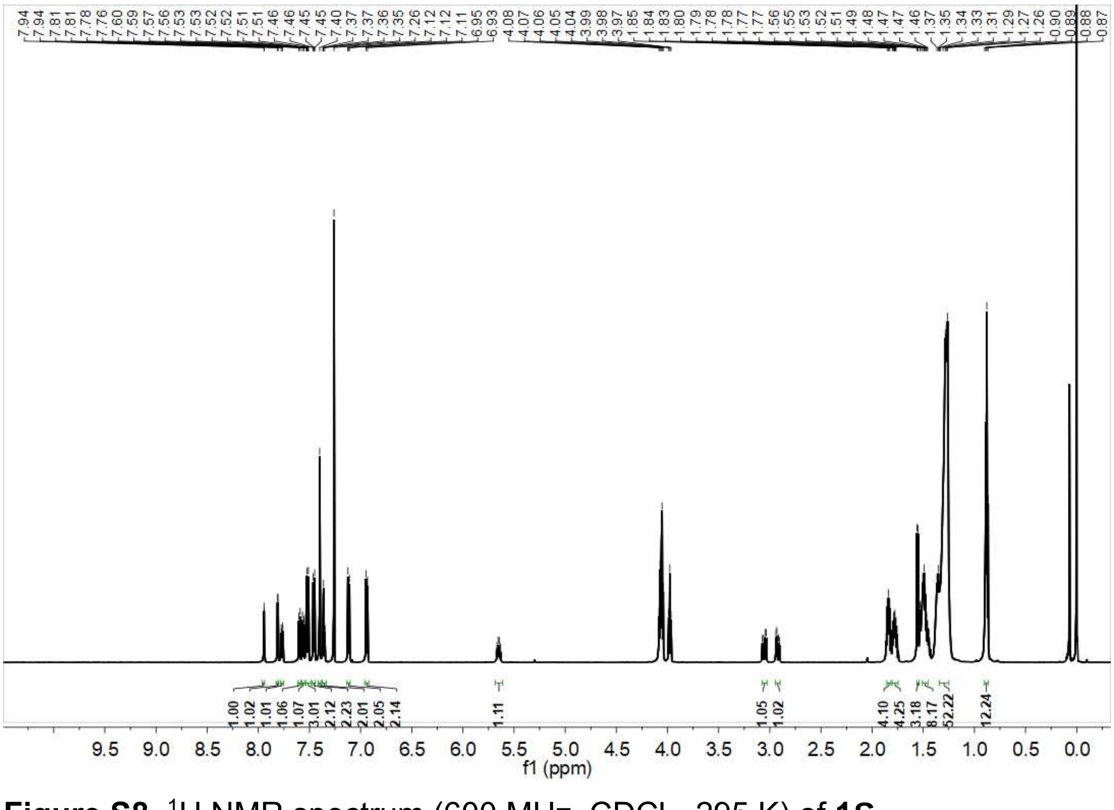

Figure S8. ^{1}H NMR spectrum (600 MHz, $CDCl_3$, 295 K) of **1*S***.

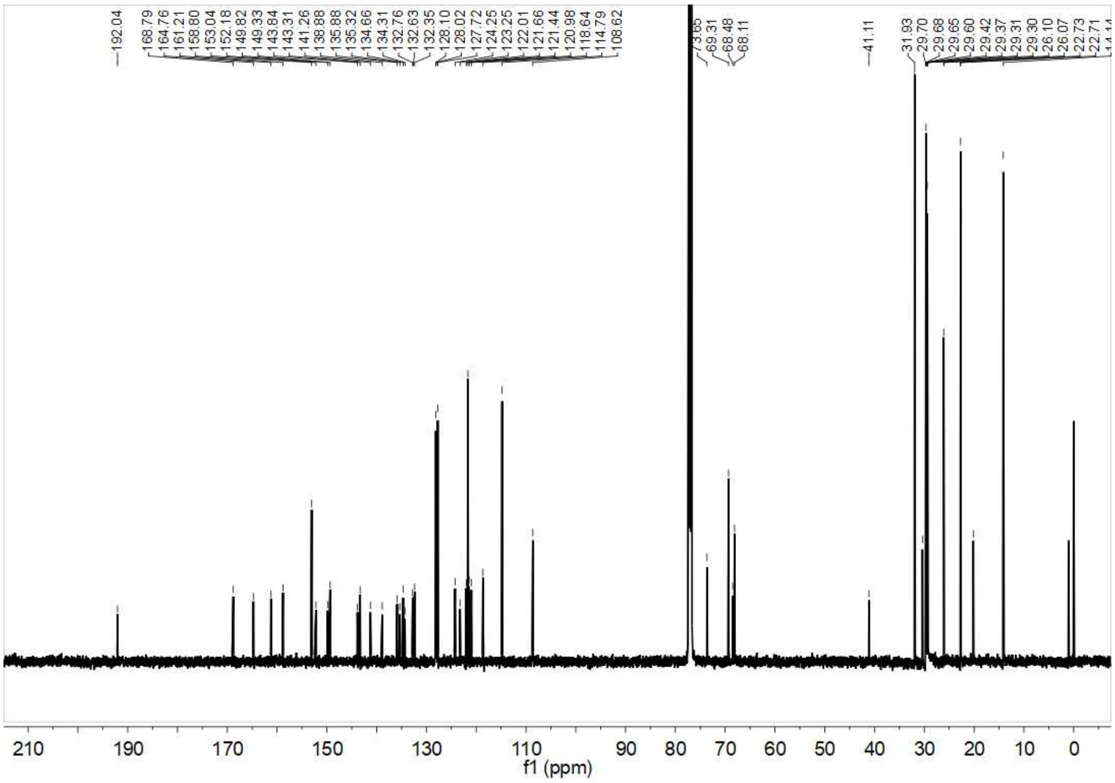

Figure S9. ^{13}C NMR spectrum (150 MHz, $CDCl_3$, 298 K) of **1*S***.

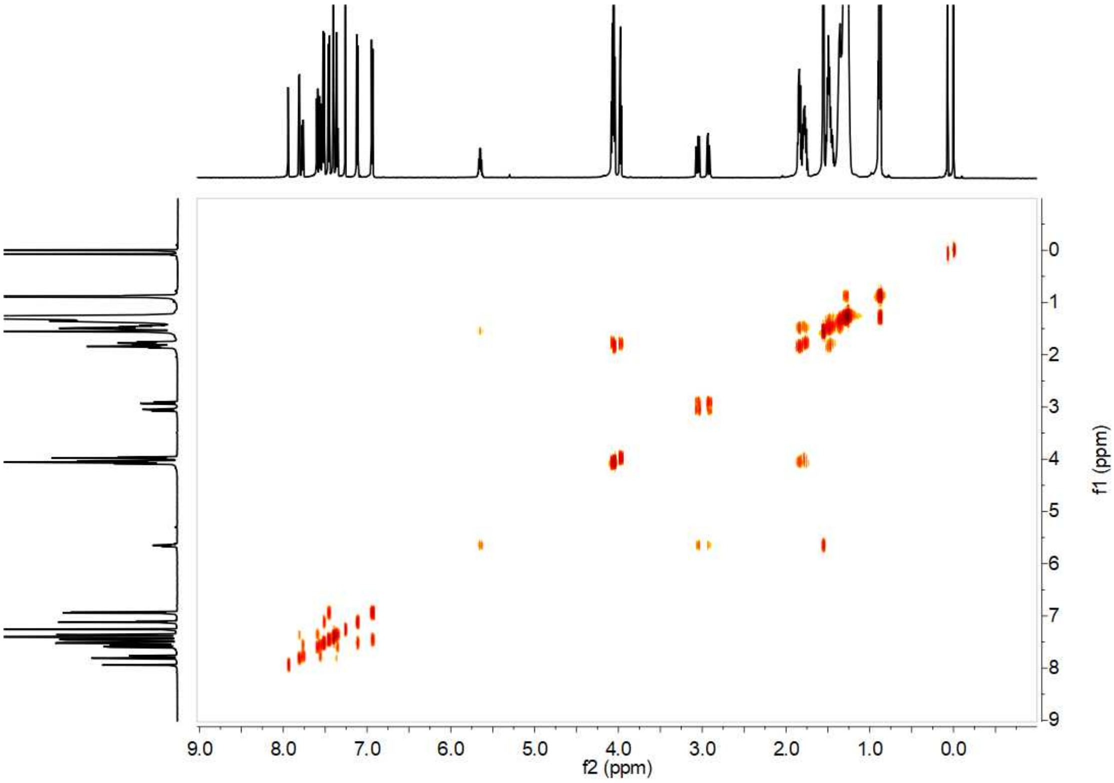

Figure S10. COSY spectrum (150 MHz, $CDCl_3$, 298 K) of **1*S***.

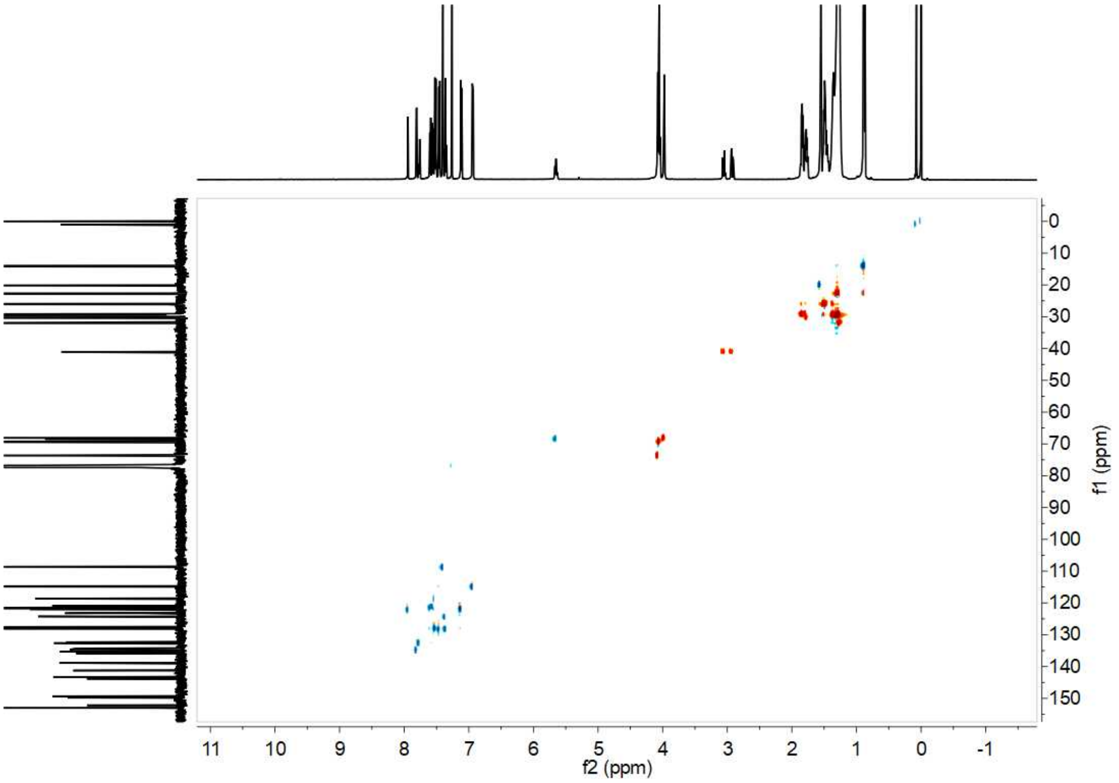

Figure S11. HSQC spectrum (150 MHz, $CDCl_3$, 298 K) of **1*S***.

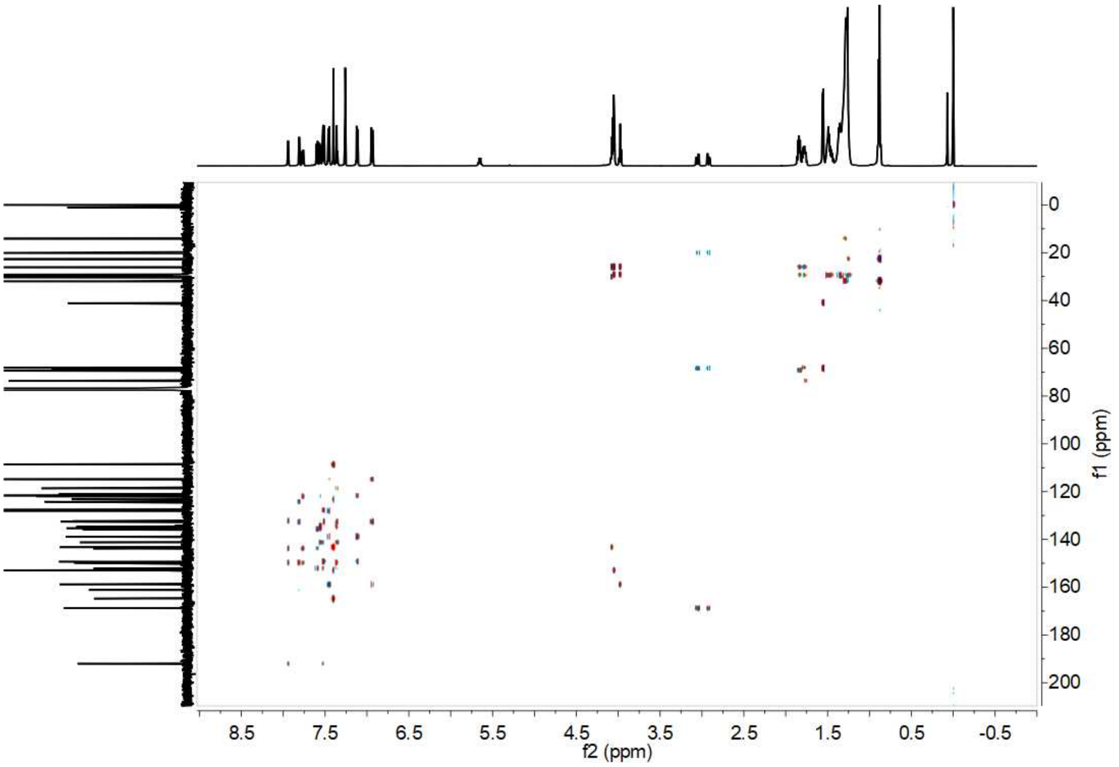

Figure S12. HMBC spectrum (150 MHz, $CDCl_3$, 298 K) of **1*S***.

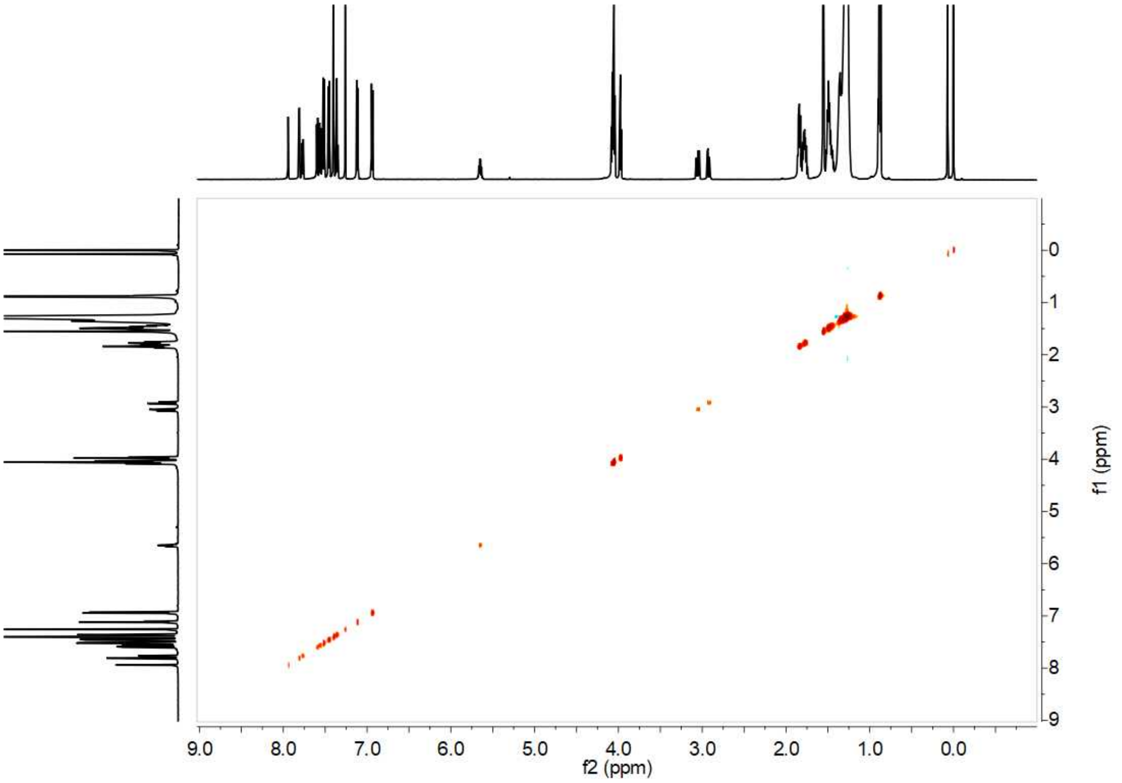

Figure S13. NOSY spectrum (150 MHz, $CDCl_3$, 298 K) **of 1*S*.**

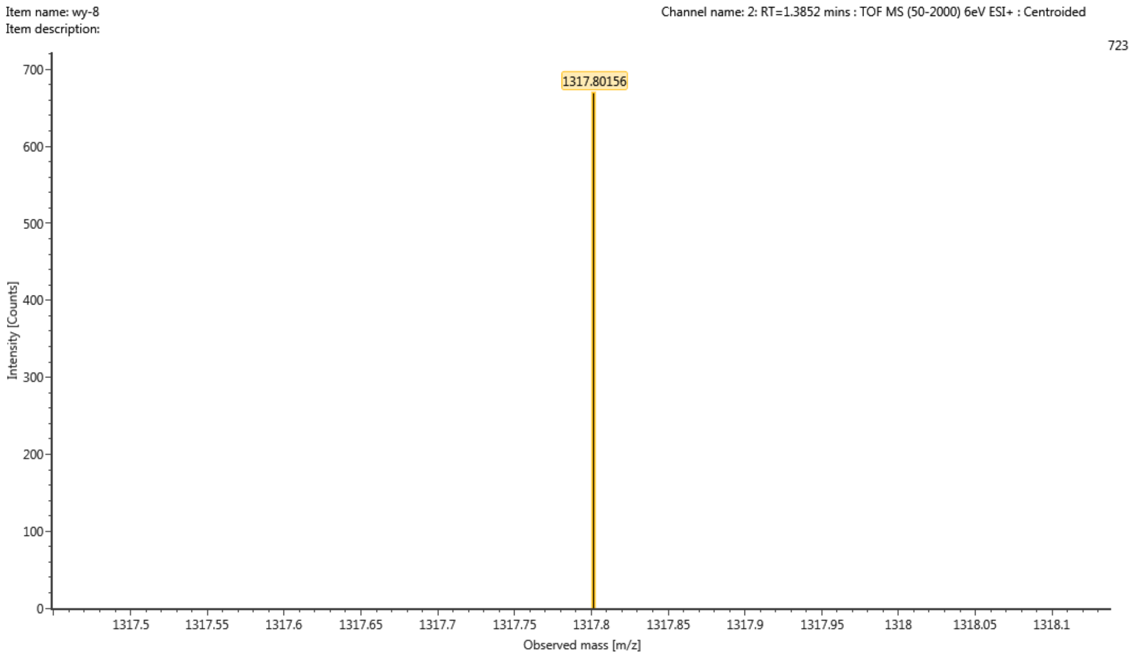

Figure S14. ESI-HR-MS spectrum of **1*S***.

S5.3 Synthesis of 1*R* compound

The same method and quantities as for ***S*** compound. It was obtained ***R*** as a yellow solid (961.26 mg, 73 %). **^{1}H NMR** (600 MHz, $CDCl_3$) δ 7.94 (d, *J* = 1.3 Hz,

1H), 7.81 (d, *J* = 3.9 Hz, 1H), 7.77 (dd, *J* = 7.8, 1.7 Hz, 1H), 7.59 (d, *J* = 8.0 Hz, 1H), 7.56 (d, *J* = 7.8 Hz, 1H), 7.54 – 7.49 (m, 3H), 7.46 (dd, 2H), 7.40 (s, 2H).7.38 – 7.34 (m, 2H), 7.12 (d,d, 2H), 6.94 (dd, 2H), 5.65 (m, *J* = 12.6, 6.3 Hz, 1H), 4.06 (m, 6H), 3.98 (t, *J* = 6.6 Hz, 2H), 3.05 (dd, *J* = 15.5, 7.7 Hz, 1H), 2.92 (dd, *J* = 15.5, 5.4 Hz, 1H), 1.88 – 1.81 (m, 4H), 1.74-1.80 (m, 4H), 1.56 (d, *J* = 6.3 Hz, 3H), 1.44-1.52 (m, 8H), 1.40 – 1.21 (overlap, 52H), 0.87-0.90 (m, 12H). **^{13}C NMR** (150 MHz, $CDCl_3$) δ 192.0, 168.8, 164.8, 161.2, 158.8, 153.0, 152.2, 149.8, 149.3, 143.8, 143.3, 141.2, 138.9, 135.9, 135.3, 134.7, 134.3, 132.8, 132.6, 132.3, 128.1, 128.0, 127.7, 124.2, 123.2, 122.0, 121.7, 121.4, 121.0, 118.6, 114.8, 108.6, 73.6, 69.3, 68.5, 68.1, 41.1, 32.0, 31.9, 30.4, 29.8-29.6, 29.4 – 29.3, 26.09, 26.06, 22.72, 22.70, 20.1, 14.1. **ESI-HR-MS**: *m/z* calcd. for $C_{83}H_{112}O_{11}SNa^+$, ([M]+Na$^+$). 1339.78176, found 1339.78482. **EA**: calcd. for $C_{83}H_{112}O_{11}S$: C 75.65 %, H 8.57 %, S 2.43 %, found: C 75.66 %, H 8.55 %, S 2.44 %.

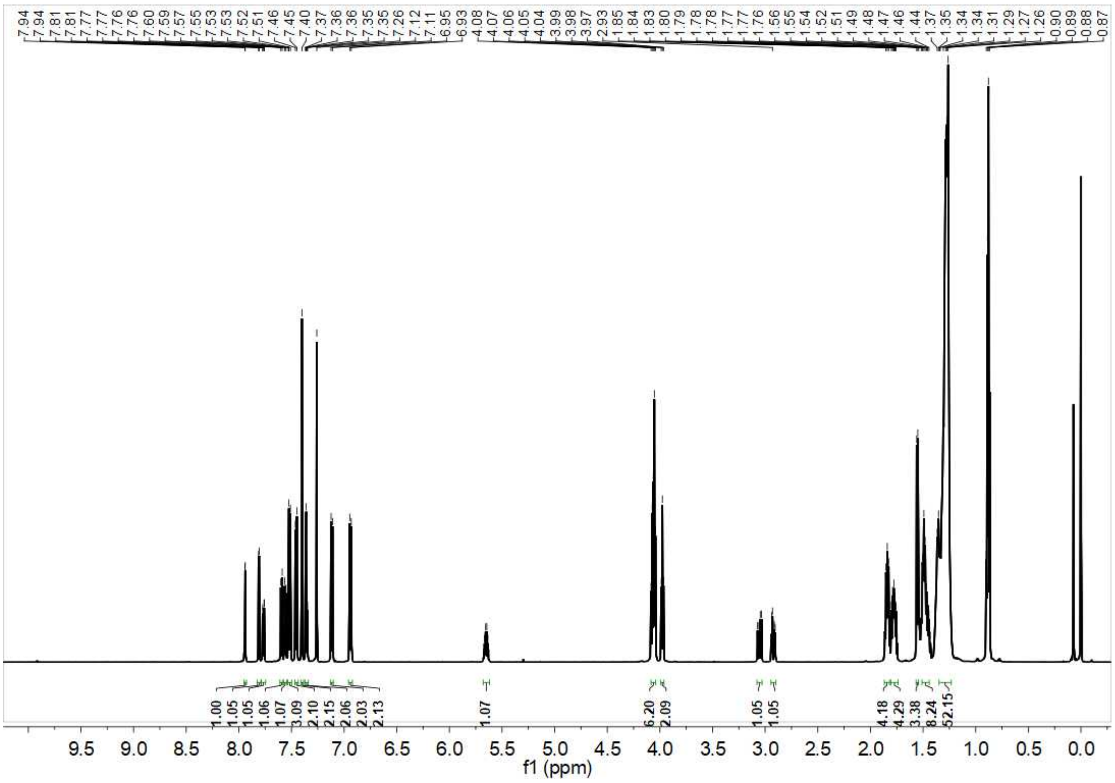

Figure S15. ^{1}H NMR spectrum (600 MHz, $CDCl_3$, 295 K) of **1*R***.

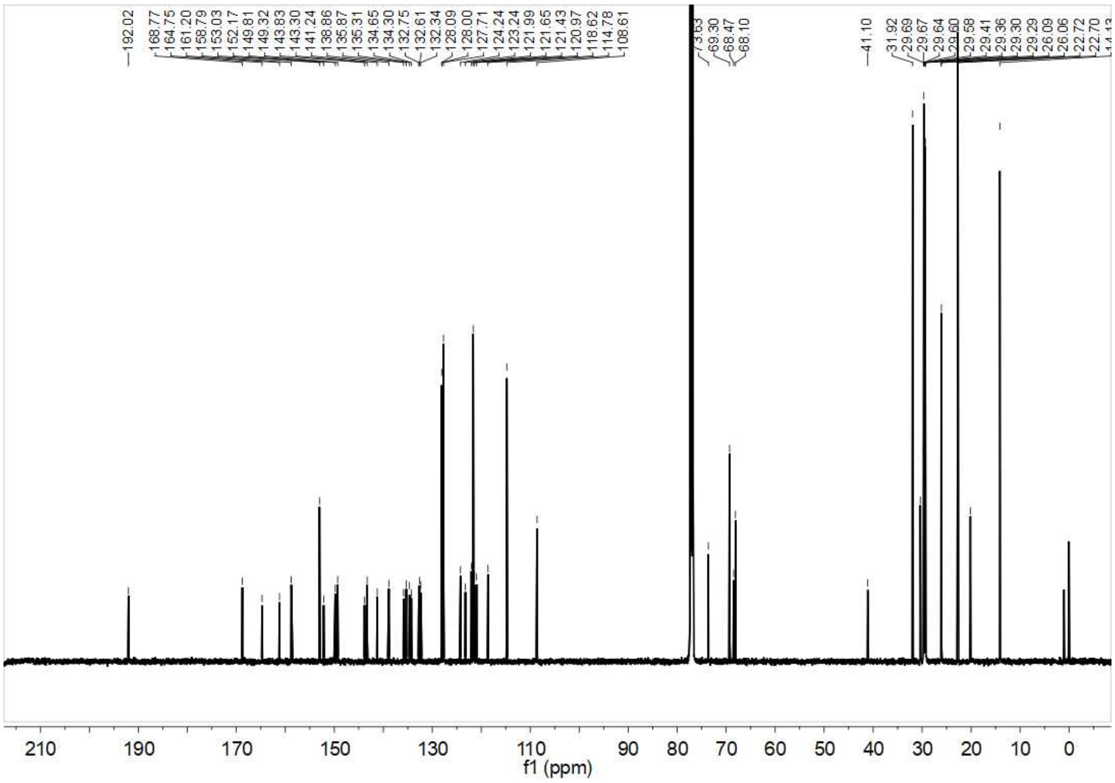

Figure S16. ^{13}C NMR spectrum (150 MHz, $CDCl_3$, 298 K) of **1*R***.

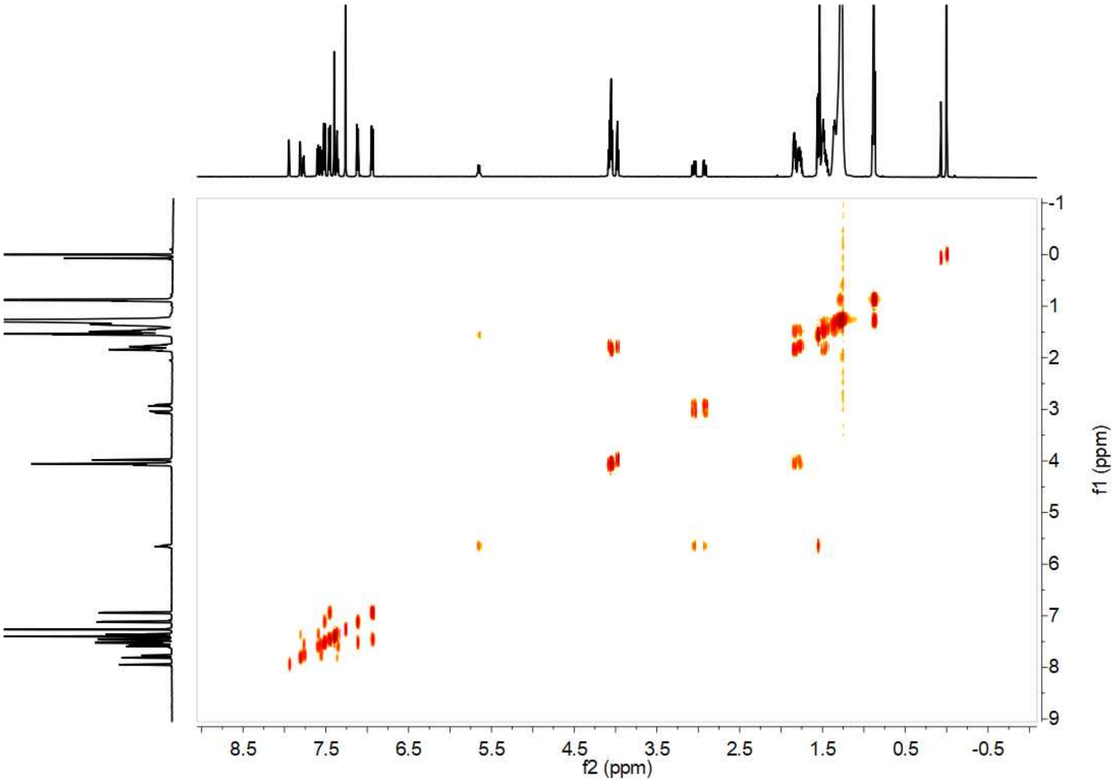

Figure S17. COSY spectrum (150 MHz, $CDCl_3$, 298 K) of **1*R***.

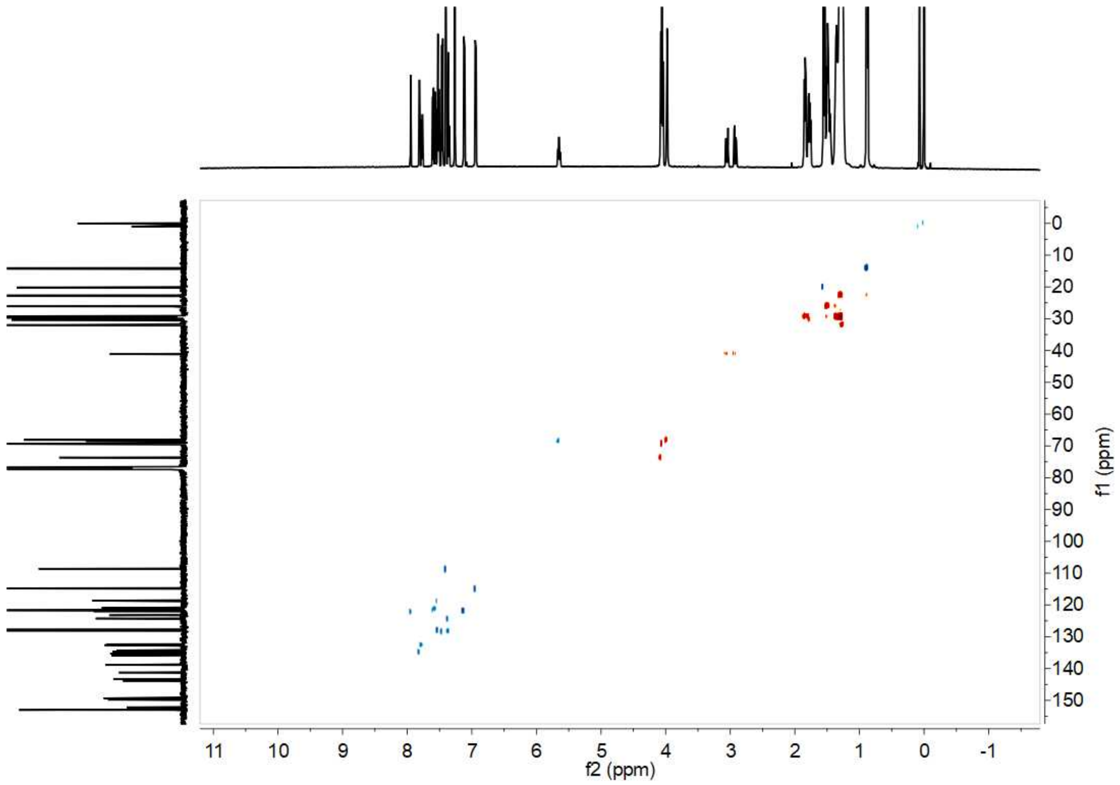

Figure S18. HSQC spectrum (150 MHz, $CDCl_3$, 298 K) of **1*R***.

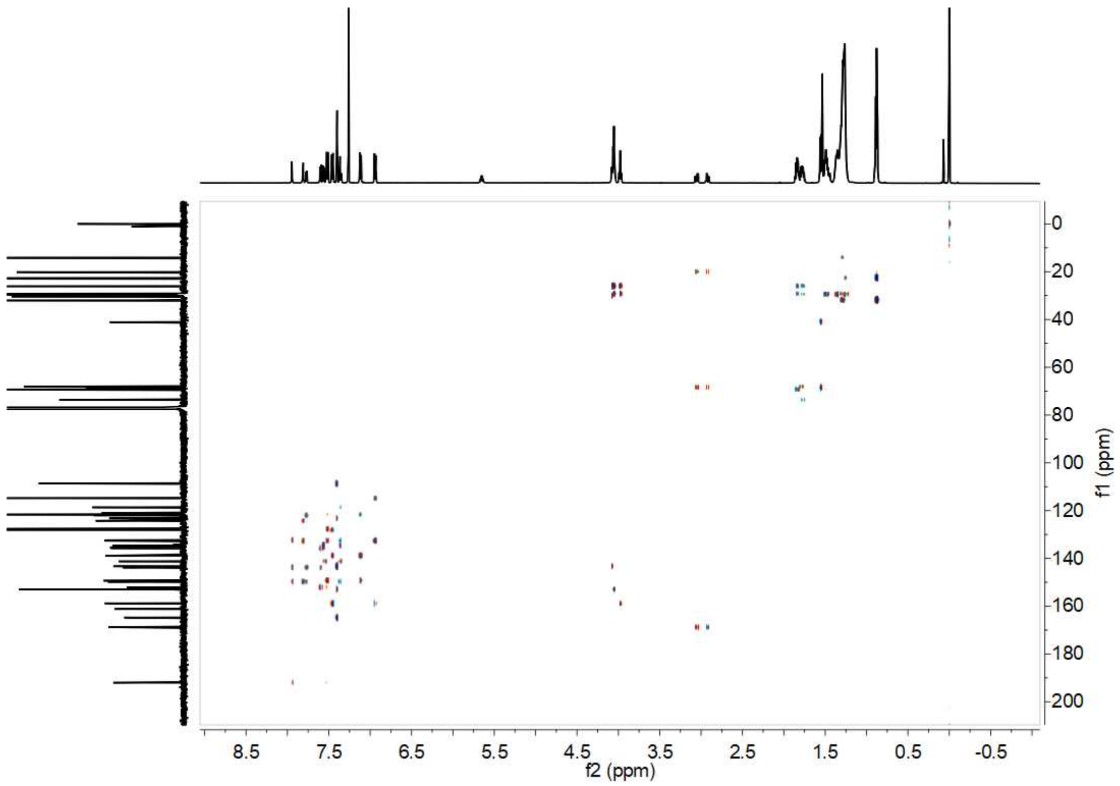

Figure S19. HMBC spectrum (150 MHz, $CDCl_3$, 298 K) of **1*R***.

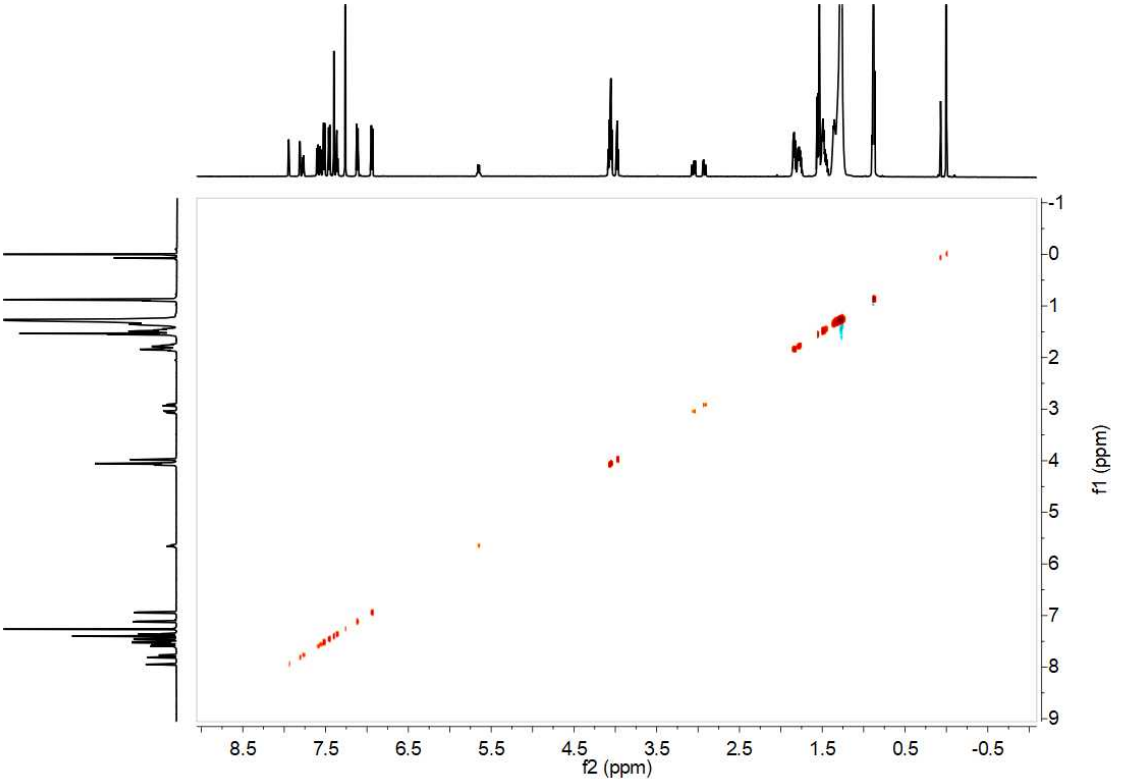

Figure S20. NOSY spectrum (150 MHz, $CDCl_3$, 298 K) of **1*R***.

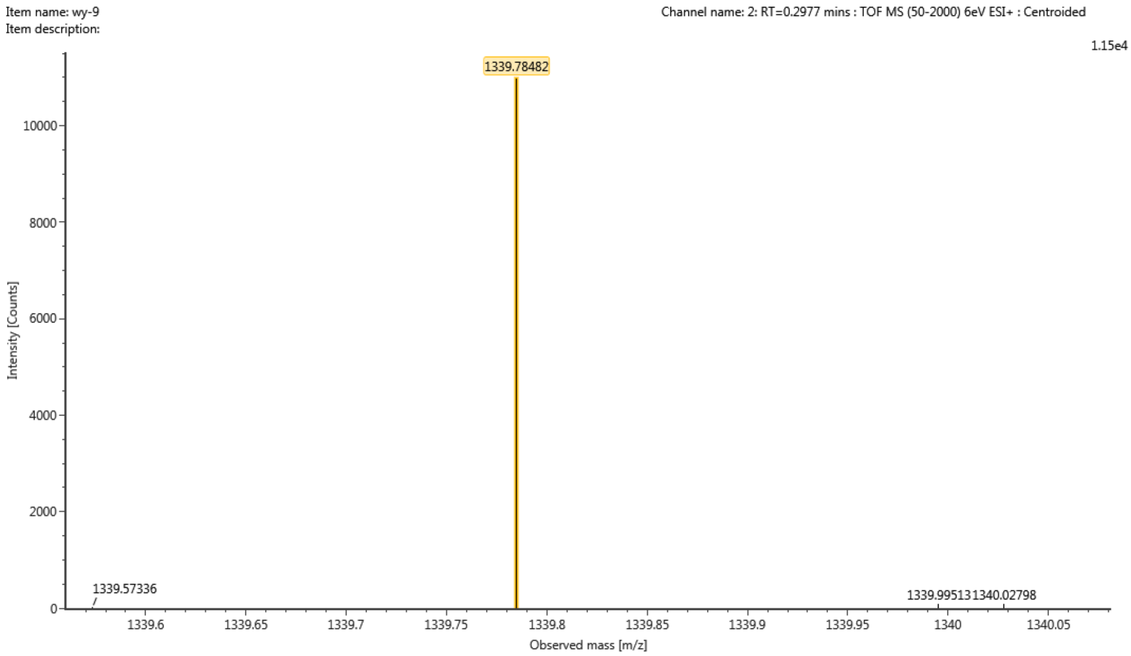

Figure 21. ESI-HR-MS spectrum of **1*R***.

S6. References

[S1] Kuroda, R.; Berova, N.; Nakanishi, K.; Woody, R. W. “Solid-state CD: application to inorganic and organic chemistry,” in Circular dichroism: principles and applications. 2nd ed.; Wiley-VCH, New York, 2000, p. 912.

[S2] X. Zeng, L. Cseh, G. H. Mehl, G. Ungar. *J. Mater. Chem.* **2008**, *18*, 2953-2961.

[S3] A. Alama, S. Tsuboib, *Tetrahedron*. **2007**, *63*, 10454-10465.

[S4] Y. Wang, Y.-X. Li, L. Cseh, Y.-X. Chen, S.-G. Yang, X.-B. Zeng, F. Liu, W. Hu, G. Ungar. *J. Am. Chem. Soc.* **2023**, *145*, 17443−17460.

[S5] L. Cseh, G.H. Mehl, *Rev. Roum. Chim.*, **2016**, *61(2)*, 125-130.
